# Role of the neurotransmitter-receptor pathway in T-cell tumor immunology and cancer immunotherapy: Neurotransmitter-receptor pathways in T-cell tumor immunology

**DOI:** 10.3724/abbs.2025216

**Published:** 2025-11-26

**Authors:** Mingyu Fan, Xiang Zhao

**Affiliations:** Key Laboratory of Multi-Cell Systems Shanghai Institute of Biochemistry and Cell Biology Center for Excellence in Molecular Cell Science University of Chinese Academy of Sciences Chinese Academy of Sciences Shanghai 200031 China

**Keywords:** T cell, tumor microenvironment, neurotransmitter, receptor signaling, cancer immunotherapy, immune checkpoint blockade, neuroimmune crosstalk, immunometabolism

## Abstract

This review synthesizes how neurotransmitters—including glutamate, acetylcholine (ACh), γ-aminobutyric acid (GABA), serotonin (5-HT), and catecholamines—modulate T-cell immunity in the tumor microenvironment through activation, differentiation, trafficking, and checkpoint dependence. Glutamate amplifies T-cell receptor signaling but is counterbalanced by tumor-derived glutamate export. Cholinergic pathways exert dual effects through nicotinic and muscarinic receptors, whereas GABA generally imposes metabolic and signaling brakes that favor regulatory programs. Serotonin shows spatial divergence—suppressing peripheral responses but enhancing intratumoral cytotoxicity—and chronic β-adrenergic stress dampens effector function and limits immunotherapy efficacy. Advances in spatial multi-omics, single-cell profiling, and neuromodulation will help discover new targets across these axes. This review provides mechanistic insights and translational implications, highlighting emerging strategies such as glutamate receptor, metabotropic glutamate receptor 4 (mGluR4) or xCT (SLC7A11) inhibition, receptor subtype modulation, and β-blockade. Integrating neurotransmitter-receptor targeting with checkpoint inhibitors or cell therapies may improve the depth and durability of cancer immunotherapy.

## Introduction

T cells are central effectors of the adaptive immune system and are essential for maintaining immune homeostasis and orchestrating antitumor immune responses [ 1, 2] . T cells are broadly classified into cytotoxic T lymphocytes (CD8 ^+^ T cells), which eliminate cancer cells primarily through perforin- and granzyme-mediated killing, and helper T cells (CD4 ^+^ T cells), which secrete cytokines to shape the immune microenvironment, promote immune-cell activation, and sustain antigen-specific responses [ 3– 5] . Studies in tumor immunology have shown that T cells play a pivotal role in recognizing and clearing malignant cells, whereas cancer cells evade immune elimination by upregulating immune checkpoints, secreting immunosuppressive factors, and recruiting regulatory T cells (Tregs) [ 6, 7] .

Neurotransmitters are chemical signaling molecules released by neurons and other cell types. They include classical small-molecule transmitters—such as acetylcholine, γ-aminobutyric acid, and glutamate—and monoamines ( *e*. *g*., dopamine and 5-hydroxytryptamine/serotonin), as well as peptide transmitters. Their functions extend beyond synaptic signaling within the nervous system to important roles in immune regulation [ 8– 10] . Growing evidence indicates that immune cells, including T cells, also express neurotransmitters and their receptors and can modulate their proliferation, differentiation, and effector functions through these pathways. This phenomenon underpins a fundamental axis of neuro-immune crosstalk [ 11, 12] . There are bidirectional signaling pathways between the nervous system and immune system: the nervous system modulates peripheral inflammation and immune responses via the vagus nerve, the sympathetic nervous system, and associated neurotransmitters, whereas the immune system can influence central nervous system activity through cytokines and other mediators [ 13– 15] . Within the tumor microenvironment, nerve fibers can innervate tumor tissue and release specific neurotransmitters that shape immune-cell function and may directly promote tumor-cell proliferation and metastasis [ 16– 18] . For example, norepinephrine released by sympathetic nerves can suppress the antitumor activity of CD8 ^+^ T cells via β-adrenergic receptors, thereby accelerating tumor progression [19].

**Figure 1:**
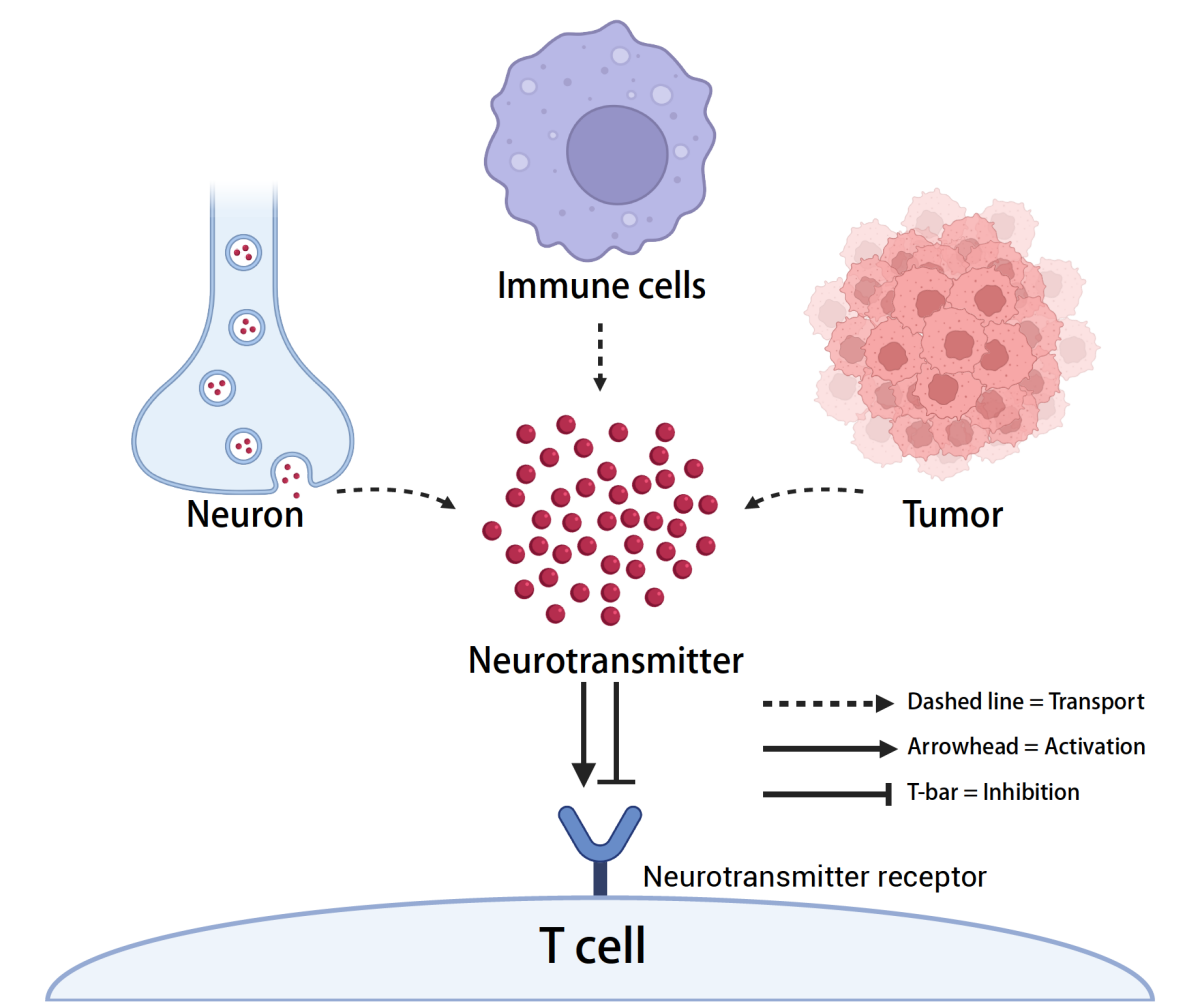
Figure 1 Neurotransmitter-mediated modulation of T-cell function within the tumor microenvironment Neurons, tumor cells, and certain immune cells release neurotransmitters into the tumor microenvironment. These neurotransmitters bind to specific neurotransmitter receptors expressed on T cells, thereby influencing T-cell activity. This process can lead to either the activation or inhibition of T-cell responses, ultimately impacting antitumor immunity. The dashed lines represent neurotransmitter transport, the solid arrows indicate activation, and the T-bar lines denote inhibition.

Accordingly, interrogating the “neurotransmitter-receptor-T-cell” axis to elucidate neuro-immune-tumor interactions not only reveals new routes of immune evasion but also identifies potential intervention targets for cancer immunotherapy. This review synthesizes current advances, summarizes the mechanisms by which neurotransmitter–receptor signaling shapes T-cell–mediated tumor immunity and cancer immunotherapy, and discusses prospects for translational application ( Figure 1).

## Glutamate

Within the tumor microenvironment (TME), glutamate (Glu), the most abundant excitatory neurotransmitter, is increasingly recognized to reshape T-cell fate and antitumor immunity along three axes: receptor, transport, and metabolism [ 20, 21] . First, T cells intrinsically express multiple glutamate receptors, and receptor signaling is functionally coupled to the T-cell receptor (TCR). Second, many cancer cells and selected myeloid immune cells ( *e*. *g*., dendritic cells and myeloid-derived suppressor cells) export glutamate via system xc ^-^ ( *SLC7A11*/xCT), which exchanges extracellular cystine for intracellular glutamate. This elevates local glutamate levels, rewires the relevant receptor pathways, and shifts the immune balance toward a suppressive state [ 22, 23] . Third, glutamate production and utilization within T cells influence effector–memory differentiation trajectories, thereby determining the quality and durability of responses to immunotherapy [ 24– 26] .

Recent studies have revealed that glutamate receptors participate in T-cell activation not only as passive metabolic sensors but also as integral signaling components. In CD8 ^+^ T cells, ionotropic and metabotropic glutamate receptors can physically and functionally associate with the T-cell receptor (TCR) complex, amplifying proximal activation cascades such as Ca² ^+^ influx and MAPK and NFAT signaling. Disruption of this interaction attenuates clonal expansion and cytotoxic activity *in vitro* and *in vivo*, indicating that glutamate receptor signaling contributes to the co-stimulatory layer of T-cell activation and may represent a pharmacologically tractable axis for immunomodulation [27]. In line with this, additional studies reported the upregulation of both ionotropic and metabotropic glutamate receptors (iGluRs and mGluRs) in tumor-infiltrating T cells. Antagonizing these receptors reduces CD69/CD25 expression, dampens Lck/Akt and Ca² ^+^ signaling, and decreases cytotoxicity, supporting the use of receptor agonists or pathway enhancement as sensitization strategies [27].

In specific pathological contexts, receptor expression on the host or tumor side can modulate antigen-specific T-cell responses in a non-cell-autonomous manner. For example, overexpression of metabotropic glutamate receptor 1 (mGluR1) in melanocytes suppresses the function of gp100-specific CD8 ^+^ T cells [28]. From a longer-term perspective of receptor plasticity, human T cells express the functional α-amino-3-hydroxy-5-methyl-4-isoxazolepropionic acid (AMPA)-type receptor GluR3, and low concentrations of glutamate are sufficient to trigger adhesion and chemotaxis [29]. However, following sustained TCR activation, GluR3 undergoes granzyme B-mediated proteolytic downregulation from the plasma membrane, creating a negative feedback mechanism that dynamically modulates T-cell responsiveness to glutamate signaling [30]. At the level of immune synapses, dendritic cells (DCs) can release glutamate via system x _c_^–^. Initially, signaling through T-cell mGluR5 moderately restrains early activation; subsequently, during a phase of TCR-driven mGluR1 upregulation, the same pathway shifts to promote proliferation and Th1-type cytokine secretion. This dynamic switch in mGluR signaling contributes to an additional layer of immune regulation that aligns with the emerging concept of a “fourth signal” in T-cell activation, integrating neurochemical cues into the canonical three-signal framework [ 22, 25, 31] .

Within the tumor microenvironment (TME), the mGluR4 (GRM4) axis has been identified by multiple *in vivo* studies as an immunosuppressive hub. In murine B16, MC38, and 3LL models, genetic or pharmacologic inhibition of GRM4 markedly delays tumor progression and acts synergistically with immune checkpoint therapy [21]. Mechanistic analyses across several models further revealed that GRM4 loss releases constraints on DC maturation and antigen presentation, thereby enhancing CD8 ^+^ T-cell and NK-cell effector responses—non-neuronal evidence supporting the negative immunoregulatory role of GRM4 in the TME [24]. Collectively, these data position GRM4 as a potential dual target linking immunometabolism and receptor signaling, providing a strong translational rationale for combination immunotherapy [ 21, 24] .

In addition to GRM4, T-cell glutamate signaling differs markedly by receptor subtype and tumor context. Ionotropic AMPA/NMDA (N-methyl-D-aspartate receptor) receptors amplify proximal TCR signaling and downstream Ca² ^+^-NFAT/MAPK/mTOR cascades, whereas metabotropic mGluRs bifurcate into Gq- and Gi-coupled programs with opposing effects on cAMP-PKA and co-stimulation [ 27, 32, 33] . Among these, GRM4 acts as an immunoregulatory brake—its inhibition enhances DC priming and CD8 ^+^ effector responses and synergizes with programmed cell death protein 1 (PD-1)/PD-L1 blockade *in vivo* [ 21, 24] . Tumor-specific receptor expression further modulates these dynamics: pathological mGluR1 drives melanoma progression, whereas gliomas exhibit heterogeneous ionotropic glutamate receptor (iGluR) expression patterns [27]. This receptor–tumor heterogeneity argues for precision strategies that quantify receptor panels within both tumor and tumor-infiltrating lymphocytes (TILs), match subtype-specific modulators to the metabolic context, and combine with xCT ( *SLC7A11*) blockade to maximize co-stimulatory and antigen-presenting gains [ 22, 23, 34, 35] . These subtype-resolved frameworks provide a practical roadmap for designing receptor-guided, timing-sensitive immunotherapies.

The glutamate “donor-transporter” axis likewise reshapes the tumor microenvironment (TME). Tumor cells depend on xCT ( *SLC7A11*) to sustain antioxidant defenses and metabolic adaptation, and KRAS-driven tumorigenesis is particularly sensitive to xCT activity; activation of xCT promotes glutamate efflux, creating a local “high-glutamate” landscape [ 22, 23] . In glioblastoma, anti-VEGF (vascular endothelial growth factor) therapy induces xCT upregulation and perturbs glutamate homeostasis, thereby enhancing Treg suppressive function and driving resistance; this effect can be reversed by combination with Treg blockade [36].

In the myeloid compartment, macrophage-specific xCT deletion inhibits M2 polarization, increases CD8 ^+^ T-cell recruitment and activation, and improves immune control in lung cancer [37]. Tumor-associated macrophages (TAMs) also express the NMDA receptor (NMDAR); its activation promotes immunosuppression, whereas pharmacologic NMDAR antagonists ( *e*. *g*., MK-801 or memantine) reprogram TAMs and act synergistically with PD-1 blockade [38]. In addition, tumor-released glutamate can blunt neutrophil cytotoxicity and indirectly suppress CD8 ^+^ T-cell proliferation; riluzole, which inhibits glutamate release, restores innate immune cytotoxicity and suppresses tumors in mice [34]. Notably, the biphasic regulation mediated by dendritic cell-derived glutamate acting on T-cell glutamate receptors provides a mechanistic basis for how timing, dose, and receptor composition together determine the direction of immune responses [ 25, 31] .

Metabolism-receptor coupling further shapes T-cell trajectories. In CD8 ^+^ T cells, glutamate-oxaloacetate transaminase 1 (GOT1) is a metabolic node required for effector differentiation; its loss restricts effector function while biasing cells toward a memory fate, suggesting that steering glutamate flux via transamination can tune the effector-memory balance [26]. From the perspective of exogenous metabolic intervention, the glutamine antagonist JHU-083 selectively suppresses tumor metabolism and alleviates hypoxia-induced acidosis while inducing effector T cells to acquire a highly activated, long-lived phenotype with enhanced mitochondrial function [35]. In melanoma models, the inhibition of tumor glutaminolysis (using the GLS inhibitor CB-839) improved the efficacy of multiple T-cell-based immunotherapies [34]. In addition, IL-16 promotes Th1 polarization by inhibiting the glutamine-to-glutamate pathway in CD4 ^+^ T cells, thereby indirectly strengthening CD8 ^+^ T cells and antitumor immunity and establishing an actionable “cytokine-glutamate metabolism-T-cell function” axis [39].

These mechanistic insights are beginning to translate. Lowering extracellular glutamate with a “blood glutamate scavenger” (BGS) strategy slows tumor growth and enhances CD8 ^+^ T-cell infiltration and apoptotic signaling in models of melanoma brain metastasis [ 40, 41] . Direct AMPA receptor-mediated excitatory neuron-tumor synapses have been demonstrated by histology and electrophysiology, providing a biological rationale for the clinical testing of α-amino-3-hydroxy-5-methyl-4-isoxazolepropionic acid receptor (AMPAR) antagonists ( *e.g*., perampanel trials targeting peritumoral hyperexcitability) [42]. On the tumor side, vaccination strategies that immunologically target xCT ( *SLC7A11*) synergize with HER2-directed therapy, reduce metastasis, and induce immune-mediated suppression of tumor stem-like cells [43].

Taken together, existing evidence supports combined interventions against GluRs—particularly the inhibitory receptor GRM4—and xCT, paired with PD-1/PD-L1 blockade or cellular therapies, to achieve additive effects across three axes: releasing inhibition, enhancing co-stimulation, and reprogramming metabolism [ 21, 24, 38, 40, 42, 43] . Moreover, it is essential to account for the dynamic nature of TCR activation states and receptor repertoires ( *e*. *g*., rapid downregulation of GluR3), as well as tissue- and tumor-derived receptor heterogeneity ( *e*. *g*., pathological mGluR1 expression), to avoid provoking opposite immune effects under different timing and dosing conditions [ 27, 30, 32] .

**Figure 2:**
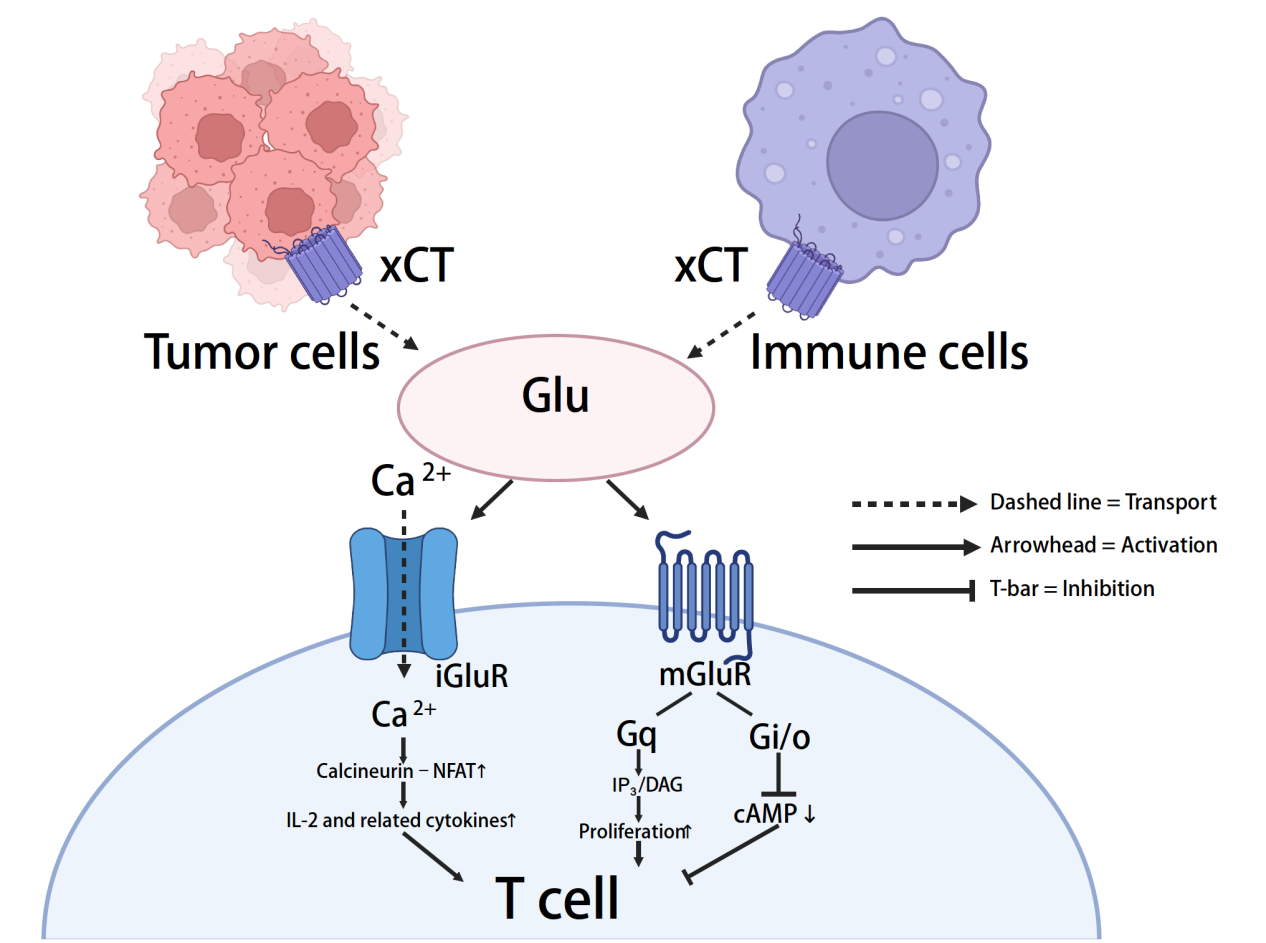
Figure 2 Glutamate release in the tumor microenvironment modulates T-cell function via glutamate receptors Tumor and immune cells export glutamate via the cystine/glutamate antiporter system xc –. Extracellular glutamate activates ionotropic and metabotropic glutamate receptors (iGluRs) on T cells, modulating signaling, cytokine production, proliferation, and effector functions.

Taken together, glutamate shapes the magnitude and quality of antitumor immunity through three coordinated axes: coupling glutamate receptor signaling to the TCR; shaping ligand availability within the TME via xCT and related pathways; and modulating T-cell effector programs through intracellular metabolic nodes. Building on these principles, multi-target combinations—centered on GRM4 inhibition, NMDAR/AMPAR modulation, xCT blockade, and reprogramming of glutamine-glutamate metabolism—constitute an emerging framework to enhance T-cell–mediated immunotherapy. These strategies warrant clinical evaluation with careful patient stratification and timing optimization [ 21– 24, 26, 36, 37, 40, 42, 44] ( Figure 2).

## Acetylcholine

Within the tumor microenvironment (TME), acetylcholine (ACh) production is not restricted to parasympathetic vagal terminals; cancer cells and immune cells also possess a complete nonneuronal cholinergic system (NNCS) [ 45– 48] . This system includes the essential enzymatic and transport machinery: cells express choline acetyltransferase (ChAT) to synthesize ACh; acetylcholinesterase (AChE) and butyrylcholinesterase (BChE) to degrade ACh; and high-affinity choline transporter 1 (CHT1) together with vesicular acetylcholine transporter (VAChT)–like components to mediate choline uptake and vesicular loading [ 49– 52] .

In studies of immune regulation in hepatocellular carcinoma (HCC), a striking observation has emerged: both HBV-associated multi-hit mouse models and human HCC specimens show clonal expansion of ChAT ^+^, which are tumor-specific CD4 ^+^ T cells [53]. Furthermore, T-cell–specific deletion of Chat weakens immune surveillance and promotes the accumulation of premalignant lesions, indicating that T-cell–derived ACh is a critical component of liver cancer immune defense [ 53, 54] . Mechanistically, ACh limits excessive TCR-induced Ca² ^+^-NFAT signaling, preventing functional imbalance and maintaining the effector-tolerance equilibrium of T cells [ 53, 55] . In addition to liver cancer, gastric cancer studies likewise reveal an autocrine ACh circuit within cancer cells [ 56, 57] . In gastric cancer cells, ACh and ChAT are upregulated and drive proliferation, providing substrates for downstream receptor signaling [56]. In addition, HCC tissues are enriched in cholinergic neurons or neurogenic features and display a targetable muscarinic receptor phenotype, supporting the presence of a “neuro–cholinergic–tumor” axis in liver cancer [58].

Acetylcholine (ACh) signals through two major receptor families: nicotinic acetylcholine receptors (nAChRs) and muscarinic acetylcholine receptors (mAChRs) [ 59– 61] .

nAChRs are ligand-gated cation channels; the α7, α5, and α9 subtypes are commonly found in tumor and immune cells [ 62– 64] . Nicotine or endogenous ACh can activate the JAK2/STAT3 axis via α7-nAChR, promoting pathways associated with tumor immune evasion [65]. In lung squamous cell carcinoma, nicotine–α7-nAChR signaling directly upregulates PD-L1 expression through STAT3-dependent transcriptional control [ 66– 68] . In melanoma, α9-nAChR mediates nicotine-induced PD-L1 expression and cross-talks with AKT/ERK and STAT3 signaling, thereby enhancing tumor cell proliferation and migration [69]. Under conditions of chronic stress or cholinergic upregulation, α5-nAChR interacts with the neuronal synaptic protein NETO2 to drive lung adenocarcinoma progression and participate in immune regulation [ 70, 71] . Recent work has shown that α5-nAChR activates a STAT3-Jab1-PD-L1 axis to mediate immune escape in lung adenocarcinoma: STAT3 binds to the *PD-L1* promoter to promote transcription while also inducing Jab1, which inhibits PD-L1 ubiquitin-mediated degradation—thus integrating transcriptional and protein stability control [68].

mAChRs (M1–M5) are G protein-coupled receptors that activate multiple downstream pathways, including the Ca² ^+^/PKC, MAPK/ERK, and PI3K/AKT pathways [ 72, 73] . In gastric cancer, the M3 receptor is functionally coupled to the epidermal growth factor receptor (EGFR); an ACh→M3R→EGFR activation cascade drives proliferation and increases sensitivity to 5-fluorouracil [ 56, 74] . In contrast, in pancreatic cancer, enhancing M1-receptor signaling can suppress the MAPK/EGFR and PI3K/AKT pathways, contract the cancer stem-like cell pool, and downregulate TNF-α-associated myeloid inflammation [ 75– 77] .

Cholinergic signaling has a complex, bidirectional regulatory pattern across T-cell subsets [ 78– 80] . Research has shown that CD8 ^+^ effector function and exhaustion are strongly affected by smoking or nicotine exposure. Through nAChRs, nicotine remodels the microRNA (miRNA) regulatory network in CD8 ^+^ T cells, inducing an exhausted phenotype and weakening anti-tumor cytotoxicity. Mechanistically, nicotine elevates miR-155 while suppressing miR-381, driving the functional exhaustion of CD8 ^+^ T cells [ 67, 81, 82] . Clinical and animal studies further indicate that nicotine rewires the FOXO1–miRNA program and perturbs transcriptional networks governing memory differentiation, suggesting that the cholinergic/nicotinic axis has lasting effects on T-cell memory [ 83– 85] . In parallel, exposure to electronic cigarettes reveals additional layers of complexity: their constituents increase PD-1 and CTLA-4 (cytotoxic T-lymphocyte-associated protein 4) expression on T cells both within and outside tumors and promote tumor metastasis, implying that exogenous nicotinic stimulation may represent a significant risk factor for immunotherapy [ 86] . In CD4 ^+^ T cells, cholinergic signaling likewise modulates the balance between T helper (Th) cells and regulatory T cells (Tregs). In a liver cancer model, a distinct ChAT ^+^CD4 ^+^T-cell subset—encompassing Tregs and a PD-1 ^+^ “dysfunctional” lineage—was identified [53]. These cells help sustain immune surveillance through cholinergic activity. Deleting the Chat gene specifically in T cells exacerbates protumor immune imbalance, which is characterized by Treg hyperactivation and impaired function of conventional CD4 ^+^ T cells [ 53, 87] .

Across multiple cancers, the cholinergic pathway is linked to immune checkpoint regulation: in melanoma, lung cancer, and colorectal cancer, cholinergic receptor expression is correlated with PD-L1/PD-L2 levels, and the upregulation of specific subtypes (α7/α9, M3) is often accompanied by increased PD-L1 expression [ 69, 88] . In clinical and preclinical studies of non-small cell lung cancer (NSCLC), noninvasive transcutaneous vagus nerve stimulation (tVNS) enhances the activation profile of tumor-infiltrating CD8 ^+^ T cells; when used alone, it is insufficient to control tumors, but it shows promise when combined with radiotherapy or immunotherapy [ 54, 86] . Lifestyle-related exposure to electronic cigarettes or nicotine increases PD-1 and CTLA-4 expression on T cells inside and outside the tumor and promotes metastasis, suggesting that exogenous nicotinic stimulation may pose a risk to immunotherapy [89].

In gastric cancer, surgical or chemical denervation and pharmacologic blockade of the M3 receptor (M3R) significantly suppress tumorigenesis and the stem cell–Wnt axis, with greater benefit when combined with chemotherapy [ 46, 47, 90] . In colorectal cancer (CRC), selective M3R inhibition or broad cholinergic blockade reduces tumor growth and downregulates the expression of immunosuppressive and cholinergic markers [ 48, 91] .

With respect to immunotherapy biomarkers, studies of patients with multiple tumor types receiving pembrolizumab have shown that an early increase in serum choline (ΔCh) is significantly associated with longer progression-free survival, indicating a measurable link between peripheral choline metabolism/cholinergic signaling and the response to immune checkpoint inhibitors (ICIs) [ 91, 92] . In adoptive cell therapy, chimeric antigen receptor T (CAR-T) cells targeting the fetal acetylcholine receptor γ subunit exhibit specific cytolytic activity in preclinical rhabdomyosarcoma models, indicating that AChR-related antigens may be druggable in select solid tumors [93].

On the muscarinic side, M3 receptor (M3R) antagonists ( *e*. *g*., darifenacin) have demonstrated peripheral selectivity and a controllable safety window in conditions such as an overactive bladder, providing pharmacologic support for a “peripheral blockade–central sparing” repurposing strategy in oncology [ 46, 88, 90, 94, 95] . In gastric and colorectal models, M3R blockade suppresses tumor growth and mitigates immunosuppression [74]. On the nicotinic side, α7-nAChR agonists ( *e*. *g*., GTS-21/DMXB-A) have shown acceptable safety in multiple early clinical studies and are supported by immunologic and anti-inflammatory rationales. However, their role in tumor immunology warrants caution to avoid pro-tumor effects mediated by nAChRs on cancer cells [ 96– 98] . Repurposing clinically approved neurotransmitter-modulating drugs requires rigorous pharmacological and translational evaluation. Peripherally selective muscarinic antagonists such as darifenacin, which exhibit minimal central nervous system penetration, have demonstrated a consistent efficacy-safety balance in phase III studies for overactive bladders, providing a rationale for potential adaptation in oncology where M3 receptor blockade suppresses tumor-promoting cholinergic signaling. The KarXT (xanomeline-trospium) combination exemplifies a central-peripheral segregation approach that may inform safer neuroimmune therapeutic designs. In addition, the nanoformulation of monoamine oxidase inhibitors (MAOIs) has been shown to reduce central toxicity and improve antitumor immune activation in preclinical studies. A structured risk assessment is recommended, emphasizing cardiovascular, psychiatric, and metabolic monitoring in combination with ICIs. [ 99– 101] .

**Figure 3:**
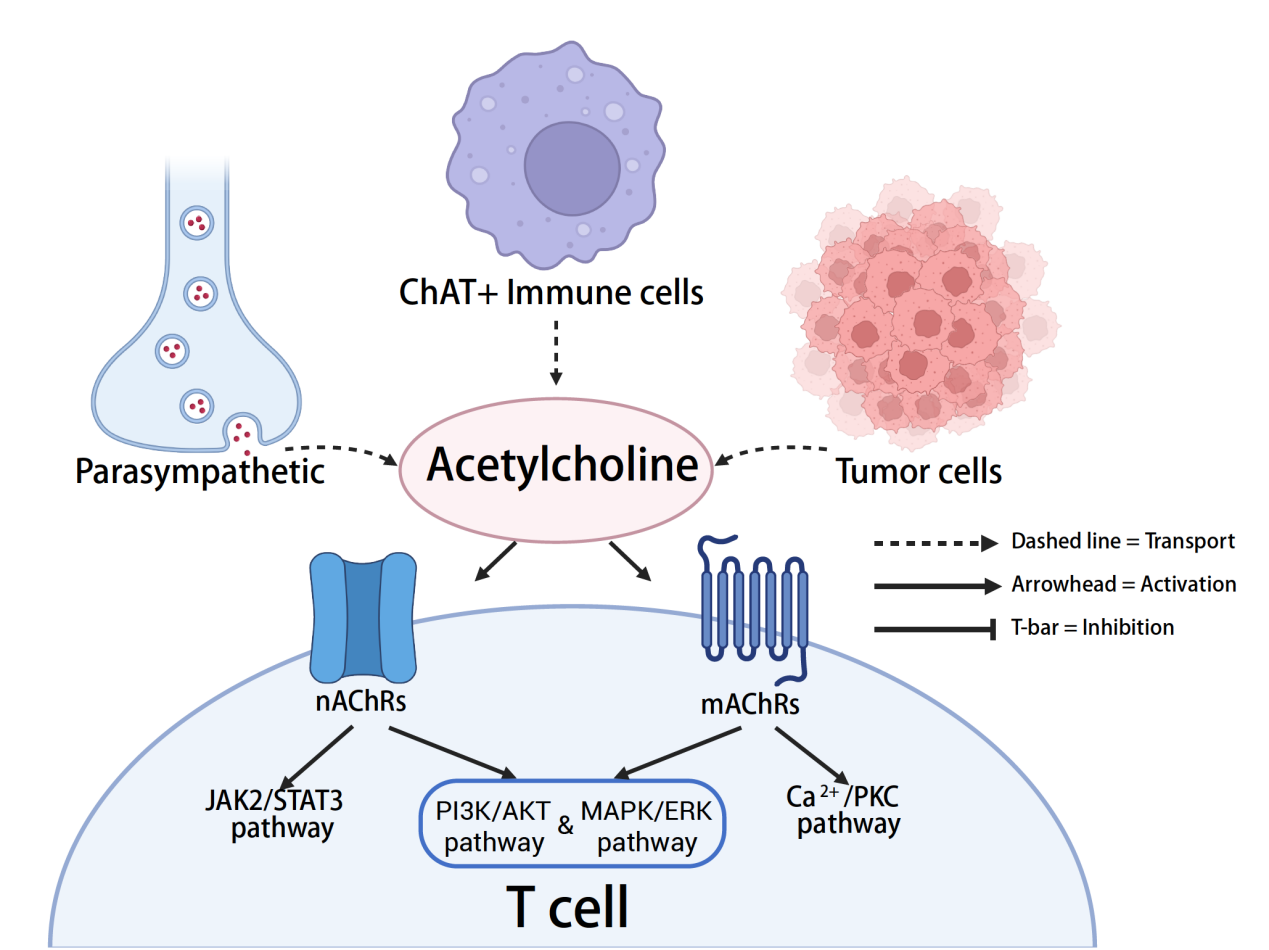
Figure 3 Cholinergic cues in the tumor microenvironment modulate T-cell function via nicotinic and muscarinic acetylcholine receptors Within the tumor microenvironment, parasympathetic neurons, tumor cells, and ChAT+ immune cells release acetylcholine (ACh). ACh engages nicotinic (nAChRs) and muscarinic (mAChRs) receptors on T cells. nAChRs primarily mediate Ca²+ influx and JAK2-STAT3 signaling, whereas mAChRs activate the PI3K-AKT, MAPK-ERK, and Ca²+/PKC pathways. These cascades collectively shape T-cell activation, differentiation, and effector function in a context-dependent manner.

These pharmacological insights highlight that rational AChR targeting requires subtype discrimination and spatial control to balance efficacy with safety [ 45, 60, 72, 73] . At the systems level, cholinergic signaling integrates the muscarinic (M1/M3 vs M2/M4) and nicotinic (α7/α9/α5) pathways with distinct immunologic outcomes [ 60, 61] . In T cells, M1/M3 (Gq) muscarinic receptors favor ERK/AKT activation and cytokine release, whereas M2/M4 (Gi) muscarinic receptors constrain cAMP; α7-nAChR may signal via JAK2-STAT3 and shape exhaustion markers. Tumor-type heterogeneity is salient: M3 dependency in gastric/colorectal cancers versus M1-linked suppression of inflammatory programs in the pancreas [ 56, 89] . Translationally, peripherally selective M3 antagonists and central/peripheral segregation designs ( *e*. *g*., combining central agonism with peripheral blockade) exemplify safety-by-design principles when targeting AChRs in oncology. For immunotherapy, avoiding non-selective exogenous agonists ( *e*. *g*., nicotine) and prioritizing subtype-selective, site-selective modulation reduces the risk of amplifying tumor-intrinsic nAChR programs that upregulate PD-L1 [ 62– 64, 68] . Overall, nonselective exogenous agonists such as nicotine should be avoided. When AChRs are expressed in both the tumor and immune compartments, receptor subtype selectivity together with site-selective delivery is essential for safety ( Figure 3).

## Gamma-aminobutyric Acid

γ-Aminobutyric acid (GABA) is the principal inhibitory neurotransmitter in the central nervous system and functions as an important modulator of immune cell activity. In the immune context, GABA signaling regulates T-cell activation, proliferation, and cytokine secretion, thereby influencing the overall intensity and quality of immune responses [102].

GABA mediates its biological functions through two major receptor families: ionotropic GABA_A receptors and metabotropic GABA_B receptors. GABA_A receptors are heteropentameric ligand-gated chloride (Cl ^–^) channels composed of various subunits (α, β, γ, δ, π, and ρ). Distinct subunit compositions confer unique conductance, desensitization kinetics, and pharmacological profiles. The π subunit (GABRP) is preferentially expressed in epithelial and tumor cells, where it contributes to membrane potential modulation, cytoskeletal organization, and intercellular adhesion. In contrast, GABA_B receptors are obligate GABA_B1/GABA_B2 heterodimers that couple to Gi/o proteins, inhibit adenylate cyclase, decrease cyclic AMP (cAMP) levels, and regulate downstream targets such as protein kinase A (PKA), GSK3β, and Ca² ^+^ or K ^+^ channels. These structural and signaling differences underpin their divergent cellular outcomes.

Foundational studies first demonstrated the presence of functional GABA_A receptors on the surface of T cells. Exogenous GABA selectively suppressed T-cell proliferation in response to antigen and anti-CD3 stimulation and inhibited delayed-type hypersensitivity *in vivo*, thereby establishing the basis of the GABA-T-cell inhibitory axis [103]. Research in both autoimmune disease models and human PBMC systems has consistently confirmed that GABA suppresses the release of proinflammatory cytokines (such as IFN-γ), reduces T-cell proliferation and metabolic activity, and consequently alleviates inflammatory responses [ 102, 104– 106] . Collectively, these findings indicate that GABA serves not only as a cross-system neuroimmune signal but also as a critical factor in shaping the tumor immune microenvironment (TME). Its influence—both in magnitude and direction—depends on factors such as its cellular origin, receptor subtype, metabolic state, and tumor type [ 107– 109] .

Extensive evidence has demonstrated that GABA_A receptor activation frequently results in protumorigenic properties. In various epithelial malignancies—including breast, pancreatic, and ovarian carcinomas—elevated GABRP expression is correlated with enhanced cell proliferation, migration, EMT, and invasion, as well as unfavorable clinical outcomes [110]. Mechanistically, GABA_A-mediated Cl ^-^ conductance influences cell volume regulation and membrane depolarization, which facilitate actin cytoskeleton remodeling and promote cellular motility. Additionally, GABA_A signaling can modulate extracellular matrix turnover and epithelial polarity, further supporting tumor invasion and metastasis.

Conversely, activation of GABA_B receptors is generally associated with anti-proliferative and tumor-suppressive effects. In non-small cell lung cancer (NSCLC), high GABA_B2 expression is correlated with improved prognosis, and receptor activation suppresses tumor cell proliferation and induces apoptosis [111]. Mechanistic studies indicate that GABA_B-dependent inhibition of the AC-cAMP-PKA cascade reactivates GSK3β, leading to reduced β-catenin activity and downregulation of proliferative- and survival-related genes. In hepatocellular carcinoma and prostate cancer, pharmacological stimulation of GABA_B signaling promotes cell cycle arrest and restrains tumor growth, supporting its inhibitory role in tumor progression. However, more recent findings suggest that the effects of GABA_B signaling are highly context-dependent, particularly within the tumor microenvironment (TME). In settings characterized by excessive GABA accumulation or reduced ABAT expression, GABA_B activation can instead stabilize β-catenin, repress CCL4 and CCL5 expression, limit CD8 ^+^/CD103 ^+^ dendritic cell infiltration, and enhance suppressive myeloid programs—collectively producing immunosuppressive rather than tumor-suppressive outcomes.

In tumor biology, aberrant accumulation of GABA is often driven by metabolic reprogramming. Many solid tumors upregulate glutamate decarboxylase GAD1 while downregulating the GABA transaminase ABAT, thereby redirecting glutamine metabolic flux toward GABA synthesis and export. This shift promotes the accumulation of GABA within the TME through autocrine and paracrine pathways [107]. Tumor-derived GABA not only acts directly on cancer cells themselves but also indirectly modulates T-cell infiltration and effector function by altering chemokine profiles and shaping myeloid cell polarization [ 107, 112] . The immune system itself can also serve as a source of GABA: activated B cells synthesize and secrete GABA, which drives monocytes to differentiate into IL-10 ^+^ anti-inflammatory macrophages, subsequently suppressing the cytotoxic activity of CD8 ^+^ T cells. In mouse models, either B-cell depletion or B-cell-specific knockout of *GAD67* reduces systemic GABA levels and enhances anti-tumor immunity [108]. Within T cells, GABA functions both as a signaling molecule and as a metabolic substrate. When ABAT is present, GABA is directed toward mitochondrial metabolism, supporting pro-inflammatory Th17 differentiation. In contrast, when ABAT is absent or inhibited, extracellular GABA accumulates and promotes iTreg differentiation, thereby shifting the overall immune response toward a more “suppressive” profile [109]. This axis has translational importance in hepatocellular carcinoma: ABAT expression is downregulated in tumor tissues, whereas forced ABAT expression reduces intra- and extracellular GABA levels, suppresses Treg differentiation, and enhances the anti-tumor activity of CD8 ^+^ T cells. Conversely, ABAT loss leads to GABA accumulation, increased Treg levels, and impaired CD8 ^+^ function [ 112, 113] . Notably, in neural tumors such as glioblastoma (GBM), GABA-related signaling and its associations with the spatial distribution of immune cell subsets and clinical prognosis have been captured at the single-cell level, further supporting the link between “origin-metabolism-immune remodeling” [114]. In lung cancer models, tumor-secreted GABA has also been shown to drive tumor-associated macrophages (TAMs) toward a pro-angiogenic and immunosuppressive phenotype [112].

T cells express functional GABA_A receptors, which, upon activation, open Cl ^-^ channels and induce changes in the membrane potential. This, in turn, enhances intracellular Ca² ^+^ influx via CRAC channels, coupling NFAT activity with the reprogramming of metabolic pathways—such as the suppression of hexokinase HK1 and glycolysis—thereby reducing metabolic activity and IFN-γ release [ 103, 115] . This effect is pronounced under physiological or low-glucose conditions (approximately 5.6 mM glucose). Insulin can upregulate the ρ2 subunit of the GABA_A receptor and amplify GABA-mediated currents, further strengthening the inhibitory effect. In contrast, under high-glucose conditions (10–16.7 mM), the suppressive effects of GABA on metabolism and cytokine production are markedly diminished or even abolished, suggesting that the peripheral metabolic status serves as a critical tuner of the GABA-T-cell axis [115]. Functionally, GABA_A receptor agonists (including benzodiazepines) suppress CD4 ^+^ and CD8 ^+^ T-cell proliferation in a dose-dependent manner, which is consistent with their rapid inhibitory effects on T-cell activation [116].

GABA_B receptors, as Gi/o-coupled receptors, further amplify immunosuppression through distinct pathways in tumor and immune cells. In cancer cells, GABA_B signaling inhibits GSK3β, stabilizes β-catenin, drives proliferation, and downregulates chemokines (such as CCL4/5), thereby reducing the recruitment of CD8 ^+^ T cells and CD103 ^+^ dendritic cells [107]. On the myeloid side, GABA_B signaling enhances arginine metabolism and NOS2 expression in granulocytic myeloid-derived suppressor cells (MDSCs), reinforcing immunosuppression and accelerating tumor progression in glioblastoma (GBM) [114]. In addition, GABA or the GABA_B receptor agonist baclofen can downregulate the E3 ligase STUB1, reducing PD-L1 ubiquitination and stabilizing its protein expression. This reshapes the immune checkpoint landscape and has significant implications for combination immunotherapy [117].

At the intersection of metabolic and immunoregulatory signaling, tumor-derived 4-acetylaminobutyric acid (4-Ac-GABA) selectively binds to the GABA_Aα3 subunit on CD8 ^+^ T cells, suppressing AKT1 phosphorylation while leaving ERK and PKA signaling unaffected. This diminishes IFN signaling without altering ERK or PKA pathways [110].

Integrating evidence from both human and murine studies, GABA generally suppresses T-cell proliferation and effector molecule production, thereby attenuating the intensity of inflammatory responses [ 102– 106, 115, 116] . At the level of migration, tumor-derived GABA downregulates CCL4 and CCL5 via the GABA_B-β-catenin axis (accompanied by reductions in CXCL9/10), markedly diminishes the intratumoral infiltration of CD8 ^+^ T cells and CD103 ^+^dendritic cells, and shapes a “non-T-cell-inflamed” TME [107]. On the stromal side, B-cell-derived GABA promotes the expansion of IL-10 ^+^ macrophages, further suppressing CD8 ^+^cytotoxicity [108]. In parallel, tumor-associated macrophages (TAMs) shift toward pro-angiogenic and immunosuppressive phenotypes under GABA signaling, jointly weakening adaptive immunity [112].

With respect to T-cell exhaustion, the 4-Ac-GABA–GABA_Aα3-AKT1 inhibitory axis coincides with reduced CD8 ^+^ T-cell responsiveness, diminished infiltration, and an increasing trend in exhaustion markers such as PD-1 and TIM-3. These findings suggest that GABAergic signaling may establish a molecular link between “metabolic restraint” and the exhaustion phenotype [110]. Moreover, receptor subunit-specific expression of GABA pathways is also associated with an immunosuppressive milieu. For example, GABRP (the GABA_A π subunit) is overexpressed in pancreatic cancer, where it promotes the accumulation of suppressive cells (Tregs, M2-like macrophages); targeting GABRP or applying GABA_A antagonists can reduce the tumor burden and restore T-cell function [111].

However, the role of GABA receptors in tumor biology is not uniformly unidirectional. In non-small cell lung cancer (NSCLC) samples, high GABA_B2 expression is correlated with improved prognosis, and *in vitro* studies have indicated that GABA can suppress cancer cell proliferation through GABA_B receptor signaling [118]. These findings underscore the context-dependent and bidirectional roles of the GABA axis, which vary according to cell type, receptor subtype, and disease context.

Evidence from human and animal tumor models supports the view of the GABA axis as a dual-pathway therapeutic target linking tumor-cell proliferation and immune suppression: inhibiting GAD1 or blocking the GABA_B receptor counteracts β-catenin-mediated pro-proliferative signaling and chemokine repression, restores CD8 ^+^ T-cell infiltration, and enhances the efficacy of anti-PD-1 therapy [107]. Upregulating ABAT or supplementing ABAT activity suppresses Treg differentiation and augments CD8 ^+^ effector function through the “metabolic clearance” of GABA, suggesting a metabolism-immunity combination strategy for hepatocellular carcinoma and other solid tumors [113]. On the other hand, pharmacologic activation of GABA_B can stabilize PD-L1 and act synergistically with anti-PD-L1 therapy, but it may also exacerbate immunosuppression or shift checkpoint dependencies, necessitating careful control of sequencing and dose [117].

**Figure 4:**
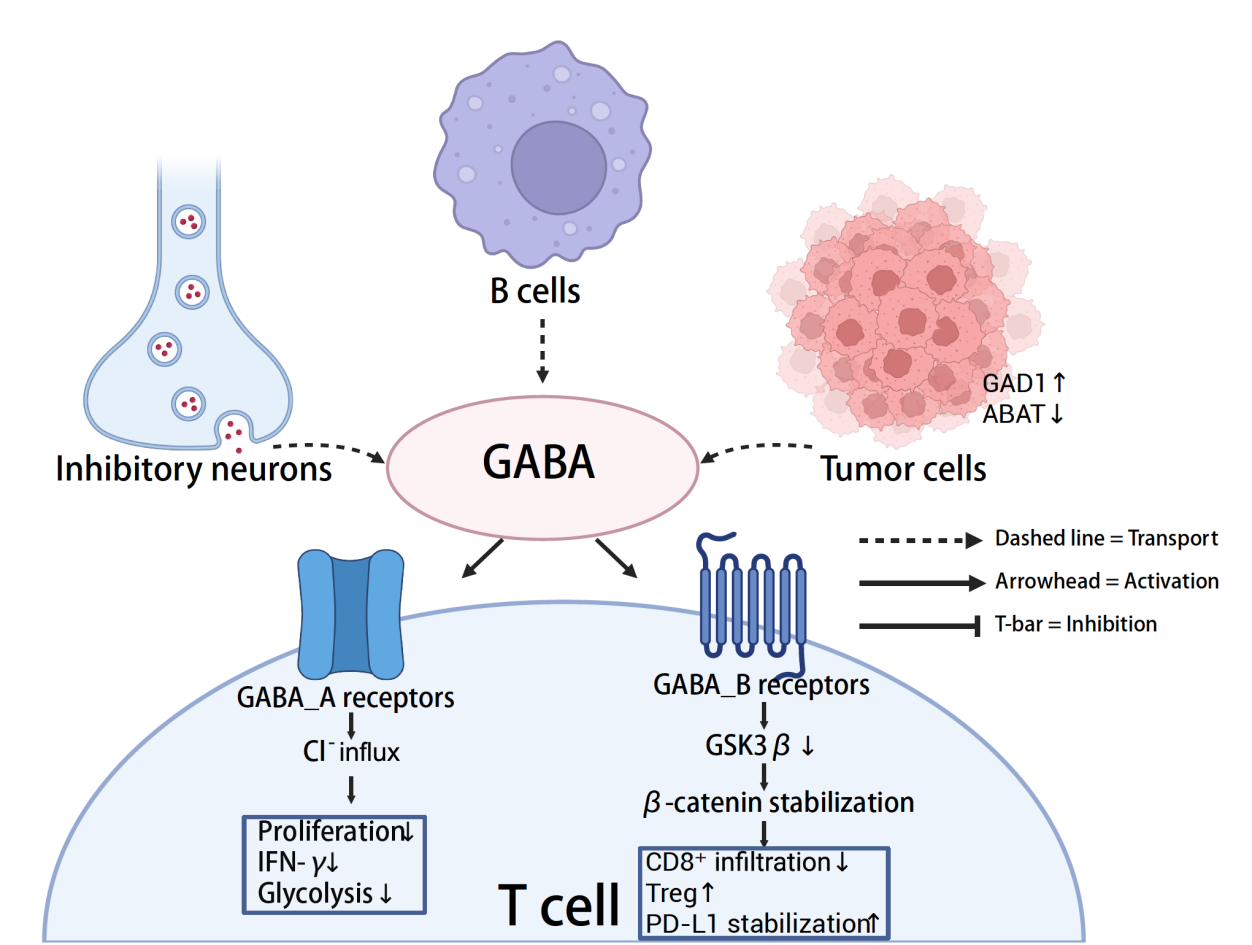
Figure 4 GABA-mediated suppression of T-cell immunity in the TME Inhibitory neurons, tumor cells, and subsets of B cells release γ-aminobutyric acid (GABA), which engages GABA_A and GABA_B receptors on T cells. GABA_A receptor-mediated Cl– influx is associated with reduced proliferation, glycolysis, and IFN-γ production, whereas GABA_B receptor signaling has been linked to β-catenin stabilization, impaired CD8+ T-cell infiltration, increased Treg frequency, and PD-L1 stabilization. Tumor cells frequently upregulate GAD1 and downregulate ABAT, promoting GABA accumulation.

With respect to GABA_A signaling, enhancing GABA_A receptor activity within cancer cells ( *e*. *g*., with benzodiazepines) induces tumor-cell membrane depolarization and promotes cell death, resulting in triple synergy with radiotherapy and anti-PD-L1 therapy [119]. However, in T cells, GABA_A receptor activation generally suppresses proliferation and IFN-γ production, indicating that these bidirectional effects—tumor-cell cytotoxicity versus T-cell inhibition—require tissue-selective optimization with respect to delivery specificity and the therapeutic window [ 115, 116] . Synthesizing these findings, strategies centered on the GABA axis can be organized along three fronts: (i) source-level control—suppressing GAD1, upregulating ABAT, or limiting B-cell-derived GABA—to reduce the GABA burden within the TME; (ii) pathway blockade—selective antagonism of GABA_B and subunit-specific modulation of GABA_A ( *e*. *g*., GABRP/α3)—to leverage key nodes spanning chemotaxis, metabolism, and exhaustion; and (iii) context-specific combinations (with anti-PD-1/PD-L1 therapies, radiotherapy, *etc*.), with consideration of metabolic status (blood glucose/insulin) and sex differences, to minimize T-cell suppression while maximizing effects on cancer cells and immunosuppressive lineages [ 105, 110, 115, 117, 119] . In addition, commonly used GABAergic medications (such as benzodiazepines and baclofen) may pose extrapolation risks in cancer patients: on one hand, they may sensitize tumors to radiotherapy and anti-PD-1/PD-L1 therapy; on the other hand, they may exacerbate peripheral immunosuppression or worsen outcomes in specific populations. Accordingly, biomarker-guided, individualized risk-benefit assessment—incorporating receptor subtypes, ABAT/GAD1 expression, and metabolic state—is warranted [ 110, 119] ( Figure 4).

## Serotonin

Serotonin (5-hydroxytryptamine, 5-HT) is a classic neurotransmitter and, at the same time, an important paracrine/autocrine signaling molecule within the immune microenvironment. T cells not only express multiple 5-HT receptors (5-HTRs) but also possess the full complement of machinery for serotonin synthesis, transport, and metabolism. Together, these features constitute a “5-HT-T-cell axis” that profoundly shapes T-cell activation thresholds, effector lineages, and patterns of tumor infiltration in tumor immunity [ 120, 121] .

Serotonin receptors and downstream signaling are diverse and context-dependent. T cells express receptor subtypes, including 5-HT1, 5-HT2, and 5-HT7, among others. In general, 5-HT1A/1B signals via Gi to lower cAMP/PKA activity and tends toward inhibitory outcomes; 5-HT2A/2B act through Gq-PLC/PKC to regulate cytokine release and migration; and 5-HT7 receptors couple to Gs to increase cAMP and promote ERK and NF-κB activation, which can enhance signaling downstream of the TCR [ 120, 122] . Classic studies have shown that blockade or loss of 5-HT1B suppresses T-cell proliferation, whereas activation of 5-HT7 enhances IL-2 and IFN-γ expression [ 122, 123] . Receptor expression and function vary with T-cell lineage and activation state: CD4 ^+^ effector subsets and regulatory T cells exhibit distinct profiles of 5-HT receptors, the biosynthetic enzyme tryptophan hydroxylase-1 (TPH1), and serotonin-metabolizing enzymes, leading to subset-specific and context-dependent effects of 5-HT [121].

The metabolism-signaling coupling of serotonin spans from receptor-mediated signaling to “protein serotonylation”. Beyond canonical receptor pathways, an atypical 5-HT effect operates in CD8 ^+^ T cells: under the mediation of transglutaminase TGM2, 5-HT covalently serotonylates the glycolytic enzyme GAPDH at Gln262, promoting its retention in the cytosol and enhancing glycolysis and effector function; meanwhile, monoamine oxidase A (MAO-A) degrades 5-HT within T cells, forming a negative regulatory loop [ 124, 125] . These findings extend the use of 5-HT from a mere membrane receptor agonist to a metabolism–posttranslational modification signal and provide actionable metabolic augmentation strategies for engineered T cells ( *e*. *g*., CAR-T cells overexpressing TPH1 or inhibiting MAO-A) [ 124, 125] .

Peripheral and intratumoral 5-HT exert opposing effects. Recent animal and human studies suggest that peripheral 5-HT—especially that derived from platelets—tends to suppress anti-tumor immunity: lowering peripheral 5-HT ( *e*. *g*., via Tph1 deficiency, TPH1 inhibition, or depletion of platelet 5-HT) slows the growth of multiple transplantable tumors, increases CD8 ^+^ T-cell infiltration, and synergizes with PD-1/PD-L1 blockade [126]. In contrast, local intratumoral 5-HT is often insufficient yet has the potential to increase effector T-cell activity: CD8 ^+^ T cells can secrete 5-HT in an autocrine manner to activate their own receptors and increase tumor killing; however, their expression of the serotonin transporter (SERT) facilitates the reuptake of extracellular 5-HT, thereby reducing its local availability and limiting effector responses. Blocking SERT with selective serotonin reuptake inhibitors (SSRIs) elevates intratumoral 5-HT, strengthens CD8 ^+^ T-cell efficacy, and acts synergistically with PD-1 blockade [127]. Consistent with this, studies in lung cancer indicate that tumor tissues are generally 5-HT-deficient; supplementing exogenous 5-HT can improve dendritic cell and CD8 ^+^ T-cell infiltration and prolong survival, suggesting that a spatially partitioned strategy—reducing peripheral 5-HT while replenishing it within tumors—may be a promising direction [128].

Targeting distinct serotonin receptor subtypes can serve as an immunotherapeutic strategy. In a murine hepatocellular carcinoma model, blocking 5-HT2A on T cells ( *e*. *g*., with *ketanserin* or *Htr2a* knockout) markedly enhances the cytotoxic phenotype of CD8 ^+^ T cells and improves *in vivo* tumor control, with further survival benefit when combined with anti-PD-L1 plus anti-VEGF-A therapy [129]. Conversely, agonizing specific receptors can enhance immunogenicity from the tumor-cell side: 5-nonyloxytryptamine (5-NL) upregulates tumor-cell MHC-I without a concomitant increase in PD-L1, thereby improving T-cell recognition and synergizing with PD-1 blockade [130]. In stress-associated tumor models, HTR1E signaling exerts protective effects by suppressing stress-driven tumor promotion, underscoring tissue and context specificity across receptor subtypes [ 128, 131] . In contrast, activation of the 5-HT1A receptor on cancer cells can upregulate PD-L1 via the autophagy/STAT3 pathway, expand Tregs, induce immune escape, and is linked to poor prognosis in depression-associated lung adenocarcinoma [132].

Building upon these mechanistic insights, serotonin signaling integrates Gi-(5-HT1), Gq- (5-HT2), and Gs-(5-HT7)-coupled programs that intersect with cellular metabolism via MAO-A activity and protein serotonylation [ 121, 122, 124, 125, 129] . A spatial dichotomy further refines this framework: reducing peripheral 5-HT ( *e*. *g*., via TPH1 inhibition or platelet depletion) enhances antitumor immunity and responsiveness to PD-1/PD-L1 blockade, whereas increasing intratumoral 5-HT—through the inhibition of SERT or MAO-A within the tumor microenvironment—can potentiate CD8 ^+^ effector function [ 125, 127, 133] . At the receptor level, targeted modulation offers complementary strategies: blockade of 5-HT2A on T cells or selective 5-HT agonists that increase tumor MHC-I expression without concurrent PD-L1 induction both provide rational combinatorial strategies with immune checkpoint inhibitors [ 129, 130] . Because 5-HT can also drive inflammation or context-dependent immune escape, such designs should be guided by biomarker panels (TPH1/MAO-A/SERT and HTR expression) and delivery systems capable of distinguishing central versus peripheral compartments [ 121, 125, 132] .

Myeloid cells and T cells communicate through the 5-HT/MAO-A axis. MAO-A not only constrains 5-HT signaling within T cells but also drives immunosuppressive polarization in tumor-associated macrophages (TAMs) through oxidative stress [125]. Inhibiting MAO-A reprograms TAMs toward a pro-inflammatory phenotype, enhances T-cell-mediated anti-tumor immunity, and synergizes with PD-1 blockade [133]. To mitigate the central nervous system side effects of MAO inhibitors (MAOIs), nanodelivery strategies ( *e*. *g*., nano-phenelzine) have increased anti-tumor efficacy in mouse models while avoiding adverse effects such as aggressive behavior [134].

**Figure 5:**
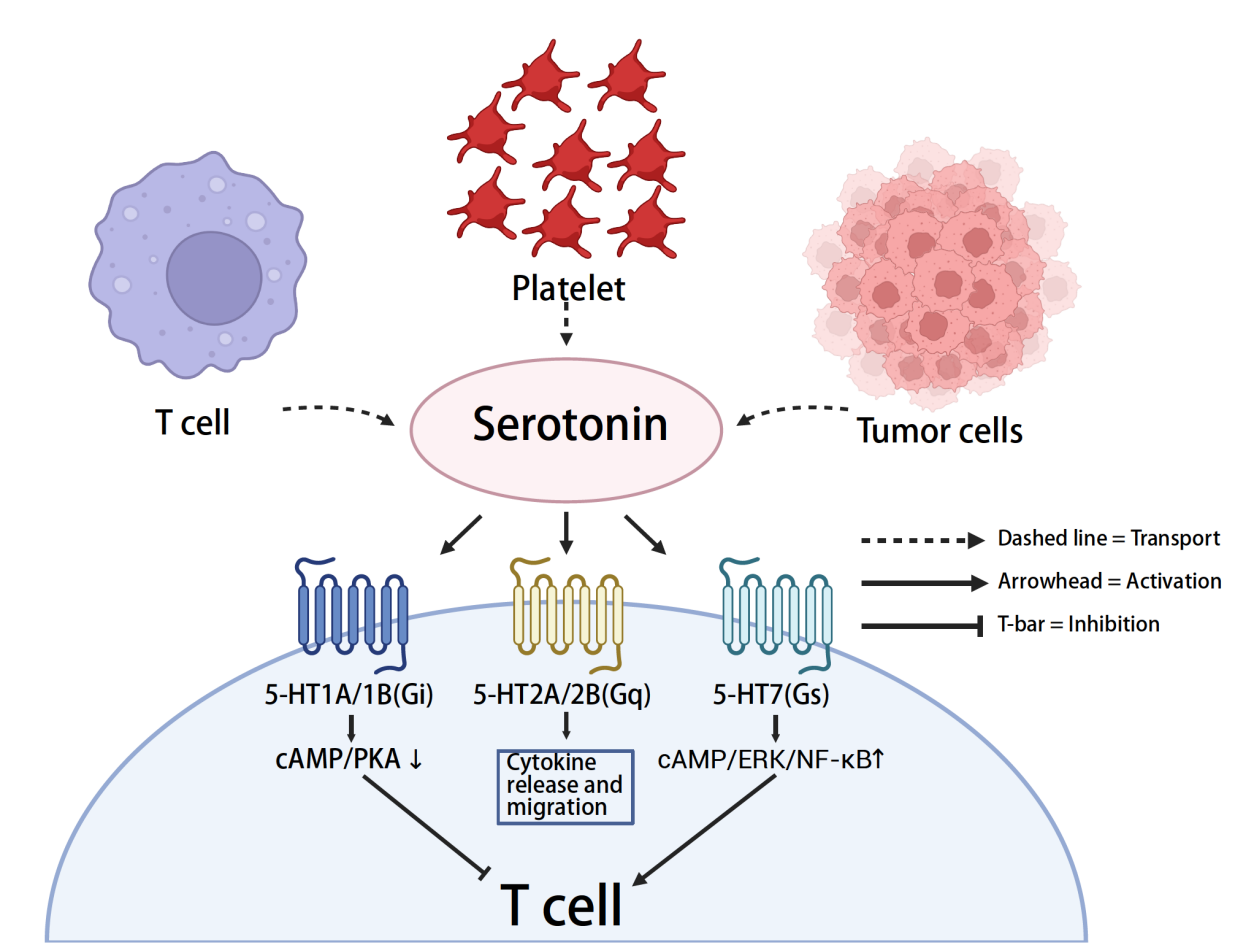
Figure 5 Serotonin signaling in the tumor microenvironment modulates T-cell function Schematic showing serotonin (5-HT) release in the tumor microenvironment from platelets (storage/release), tumor cells (uptake or synthesis), and immune compartments. 5-HT engages distinct receptors on T cells, including 5-HT1A/1B (Gi), 5-HT2A/2B (Gq), and 5-HT7 (Gs). These pathways exert both inhibitory and activating influences on T-cell function.

Multiple studies support the therapeutic potential of applying existing drugs and combination treatment strategies that target the 5-HT-T-cell axis. Selective serotonin reuptake inhibitors (SSRIs), such as fluoxetine and citalopram, exhibit anti-tumor and immunosensitizing effects across multiple models, including reversal of stress-induced kynurenine pathway activation, enhancement of tumor immunogenicity (via GLUT1 targeting and reversal of the Warburg effect), and synergy with PD-1/PD-L1 blockade [ 135, 136] . Retrospective clinical data likewise suggest that concomitant fluoxetine use is associated with improved overall survival in patients receiving PD-1/PD-L1 therapy, although prospective trials are still needed for confirmation [137]. In addition, serotonin receptor–targeting agents such as tegaserod (a 5-HT4 agonist) and 5-nonyloxytryptamine (5-NL) also have the potential to augment immune clearance [ 130, 138] . Notably, however, 5-HT can promote an inflammatory microenvironment and colorectal tumorigenesis through TIAM2S-mediated disruption of homeostasis, indicating that interventions along this axis must balance immunologic efficacy with inflammation-related safety [139] ( Figure 5).

## Catecholamines

The sympathetic nervous system (SNS) is activated by diverse stressors—including cold exposure, surgery, psychological stress, and tumor-derived stimuli—triggering the release of large amounts of norepinephrine (NE) and adrenal medulla–derived epinephrine (EPI) into the circulation. These catecholamines bind to β-adrenergic receptors (β-ARs), especially β _2_-ARs, which are highly expressed on immune cells, thereby engaging the downstream Gs-cAMP/PKA signaling axis. This pathway profoundly influences T-cell metabolism, differentiation, trafficking, effector function, and response to immunotherapy, with pronounced context and temporal dependence [ 19, 140– 142] . Under most conditions of chronic, high-level stress, this axis tends to suppress Th1 and cytotoxic T lymphocyte (CTL) activity, constituting a key neuroendocrine mechanism of tumor immune evasion [ 143– 145] . Adrenergic receptors are categorized into α- and β-families with distinct subtypes and G-protein coupling profiles: α _1_-ARs (α _1_A/α _1_B/α _1_D; Gq/11), α _2_-ARs (α _2_A/α _2_B/α _2_C; Gi/o), and β-ARs (β _1_/β _2_/β _3_; Gs) [146]. The polarity of these second-messenger pathways provides a mechanistic framework for understanding their immunological consequences within the TME. Specifically, β‑AR→Gs‑cAMP/PKA signaling generally attenuates proximal T-cell receptor (TCR) activation and aerobic glycolysis; α _2_-AR→Gi signaling reduces intracellular cAMP, thereby potentially counteracting β-adrenergic suppression; and α _1_-AR→Gq/PLC-IP _3_/DAG-Ca² ^+^/PKC signaling elicits calcium-dependent responses that vary according to the lymphocyte state and microenvironmental context. This mechanistic taxonomy elucidates why persistent β-AR activation impairs CTL function in tumors, whereas selective α _2_-AR stimulation may mitigate such immunosuppressive effects [ 146– 148] .

In tumor-bearing animal models, extensive experimental evidence has shown that sustained sympathetic activation weakens CD8 ^+^ T-cell activation and effector function, resulting in reduced efficacy of immune checkpoint inhibitors (ICIs). In a study by Bucsek *et al*. [140], housing mice at standard laboratory temperature (~22°C) induced chronic cold stress and elevated plasma NE. Through β _2_-adrenergic receptor (β _2_-AR) signaling, tumor-infiltrating CD8 ^+^ T cells (TILs) exhibit downregulation of T-bet, IFN-γ, and granzyme B (GzmB); upregulation of PD-1; loss of the effector phenotype; and a markedly blunted response to anti-PD-1 therapy. Raising the housing temperature to thermoneutrality (~30°C), administering the nonselective β-blocker propranolol, or deleting the host *β _2_-AR* gene reversed TIL dysfunction, restored cytotoxic activity, and improved survival. Similarly, in B-cell lymphoma immunotherapy, Nissen *et al*. [141] reported that chronic β-AR stimulation suppressed antigen-driven proliferation and IFN-γ secretion by CD8 ^+^ T cells, reduced their target-cell killing capacity, and ultimately weakened the efficacy of combined PD-1 and 4-1BB therapy.

The negative regulation of T cells by epinephrine (EPI) and norepinephrine (NE) involves not only signal transduction but also disruption of metabolic reprogramming. Using isoproterenol to model stress, Qiao *et al*. [142] found that β _2_-AR activation suppresses GLUT1 expression, glucose uptake, glycolysis, and mitochondrial respiration at the critical early phase of CD8 ^+^ T-cell activation, resulting in metabolic incapacitation of T cells and severely impairing their effector function. In addition, chronic β _2_-AR signaling can drive the expansion of myeloid-derived suppressor cells (MDSCs) and maintain their high suppressive activity, which is characterized by the upregulation of arginase-1 (ARG1) and PD-L1 and an increased capacity to inhibit T-cell proliferation [19]. Deletion of β _2_-AR or treatment with the nonselective β-blocker propranolol reduces MDSC survival and immunosuppressive function, effectively removing this constraint on T-cell function. Further studies have shown that chronic adrenergic stress imprints tumor-infiltrating lymphocytes (TILs) with a terminally exhausted phenotype marked by elevated expressions of inhibitory receptors (PD-1, TIM-3, and others) and low metabolic activity; β-blockade or β _2_-AR deficiency restores glycolysis, CD28 expression, and cytokine release, thereby promoting tumor control [144].

This neuroendocrine immunosuppression has important implications across multiple treatment settings. Radiotherapy depends on systemic T-cell immunity to elicit the so-called abscopal effect; Chen *et al*. [143] reported that mice under high sympathetic stress exhibited virtually no regression of distant tumors ( *i*. *e*., lacked an abscopal response). Warm housing, β-blockade, or genetic deletion of *β _2_-AR *markedly enhanced the radiotherapy-induced systemic T-cell response, and in bilateral tumor models, the growth of contralateral, non-irradiated tumors was significantly restrained. In the context of ICI therapy, extensive animal experiments and clinical analyses have consistently shown that nonselective β-blockers can enhance the efficacy of anti-PD-1 or anti-CTLA-4 antibodies through mechanisms including increased CD8 ^+^ TIL infiltration, reduced proportions of Tregs and MDSCs, and improved cytokine profiles [ 149– 151] . Notably, selective β _2_-AR blockers have shown no benefit—and may even be unfavorable—in combination with anti-PD-1 therapy, underscoring the importance of specifically targeting β _2_-AR [ 140, 152] .

The effects of β-adrenergic signaling on T cells are bidirectional and context-dependent. In chronic psychological stress models such as social isolation, Zhao *et al*. [153]reported that isolated housing elevates norepinephrine (NE) within the tumor microenvironment and enhances β _2_-AR signaling, markedly reducing CD8⁺ T-cell infiltration and IFN-γ production and accelerating tumor progression; pharmacologic blockade of β _2_-AR restores T-cell function and improves the efficacy of anti-PD-1 therapy. Surgical stress likewise suppresses CD8 ^+^T-cell and natural killer (NK) cell functions through catecholamine release, promoting postoperative metastasis, whereas perioperative β-blockade significantly reduces metastatic formation. In ovarian cancer patient samples, high intratumoral NE is associated with increased infiltration of M2-like macrophages and Tregs, diminished CD8 ^+^ T-cell activity, and poorer prognosis [154]. However, transient, low-level increases in epinephrine (EPI) can promote immunity under certain physiological conditions. For example, Miao *et al*. [155] reported that moderate-intensity exercise elevated serum EPI in murine lung cancer models; upregulated CCL5 and CXCL10 within tumors; promoted the recruitment, activation, and effector differentiation of CD8 ^+^ tumor-infiltrating lymphocytes (TILs); increased IFN-γ, TNF-α, and granzyme B (GzmB) levels; and slowed tumor growth. Low-dose exogenous EPI injections recapitulated the effects of exercise. These findings indicate that the immune consequences of EPI/NE depend on the dose, exposure duration, and microenvironmental context: high-intensity, chronic stimulation tends to be immunosuppressive, whereas low-level, acute stimulation can mobilize effector T cells.

Interventions targeting the β-adrenergic axis are under active exploration. The nonselective β-blocker propranolol has shown synergy with anti-PD-1 and anti-CTLA-4 antibodies in multiple animal models [ 140, 142, 149, 151] , and retrospective analyses in patients with melanoma and other cancers have associated its use with longer relapse-free and overall survival. In colorectal cancer, perioperative administration of propranolol combined with a COX-2 inhibitor attenuated postoperative pro-metastatic and inflammatory markers while preserving T-cell activation phenotypes [156].

Additionally, Ajmal *et al*. [157] genetically removed *β _2_-AR* from CAR-T cells, which led to increased activation (CD69, IFN-γ, and GzmB), increased proliferation, and reduced apoptosis *in vitro* and *in vivo*. These findings indicate that endogenousβ _2_-adrenergic signaling within tumor-infiltrating lymphocytes exerts a cell-intrinsic inhibitory effect on T-cell activation and persistence, thereby limiting the sustained antitumor efficacy of adoptive T-cell therapies [147]. While most studies emphasize β-AR–mediated suppression of T-cell function, α-adrenergic signaling represents a complementary and potentially actionable pathway. In particular, activation of presynaptic α _2_-ARs (Gi/o-coupled autoreceptors) reduces NE release and thereby attenuates β _2_-AR signaling on TILs. Consistent with this mechanism, α _2_-AR agonists ( *e*. *g*., clonidine and guanfacine) limit tumor growth in immunocompetent murine models (melanoma and breast cancer), increase intratumoral PD-1 ^+^CD8 ^+^ T-cell infiltration, and improve the response to anti-PD-1 therapy [147]. These observations support a model in which α _2_-AR agonism indirectly restores CTL trafficking and effector programs by mitigating β-AR-driven cAMP-PKA signaling within the tumor microenvironment without requiring generalized ablation of sympathetic input. Notably, direct α _2_-AR expression on T cells appears to be low and context-dependent, and current evidence favors a predominantly presynaptic/neuromodulatory mechanism that enhances T-cell-mediated tumor control; careful dose scheduling and safety monitoring, including cardiovascular and central nervous system assessments, are needed for clinical translation [147].

In addition to direct effects on T cells, β-AR signaling can influence T-cell trafficking and positioning by modulating blood vessels, lymphatic vessels, and the stroma. For example, Devi *et al*. [145] reported that acute β-AR stimulation induces local vasoconstriction and hypoxia, immediately blocking the trafficking of CD4 ^+^ and CD8 ^+^ T cells between tissues; oxygen supplementation or β-blockade restores their motility. Under chronic stress, NE signaling remodels peritumoral lymphatic networks, increasing permeability, promoting metastasis, and diminishing immune-cell influx—effects that are partially reversible with β-blockade [158]. In specific tumor types, β-AR signaling also mediates distinct immune evasion mechanisms. In neuroblastoma, Bruno *et al*. [159] reported that β _2_-AR signaling drives IFN-γ produced by TILs to upregulate tumor PD-L1, creating an immunosuppressive feedback loop; β _2_-AR antagonists disrupt this loop and restore T-cell efficacy. In lung adenocarcinoma, Geng *et al*. [160] reported that NE activates Wnt7A-β-catenin signaling to downregulate CXCL9 and increase adenosine levels, thereby reducing CD8 ^+^ T-cell infiltration and IFN-γ production and leading to anti-PD-1 resistance; β-AR blockade reverses this process.

Recent clinical data demonstrate that β-adrenergic blockade in combination with immune checkpoint inhibitors (ICIs) provides measurable efficacy and acceptable safety in patients with advanced malignancies [161]. In a phase I trial of propranolol plus pembrolizumab for advanced melanoma, the objective response rate (ORR) reached 78%, with propranolol administered at 30 mg twice daily as the recommended phase II dose (RP2D) [161]. The most frequent treatment-related adverse events included rash, fatigue, and vitiligo. Ongoing trials (NCT03384836 and NCT05968690) are further evaluating this combination in larger cohorts [ 162, 163] . Similarly, a prospective randomized trial (NCT07174947) is assessing the efficacy and safety of fluoxetine combined with PD-1/PD-L1 blockade in the treatment of hepatobiliary malignancies. These data collectively indicate the potential clinical importance of neurotransmitter-targeted strategies in enhancing immunotherapy efficacy while maintaining acceptable tolerability [164].

**Figure 6:**
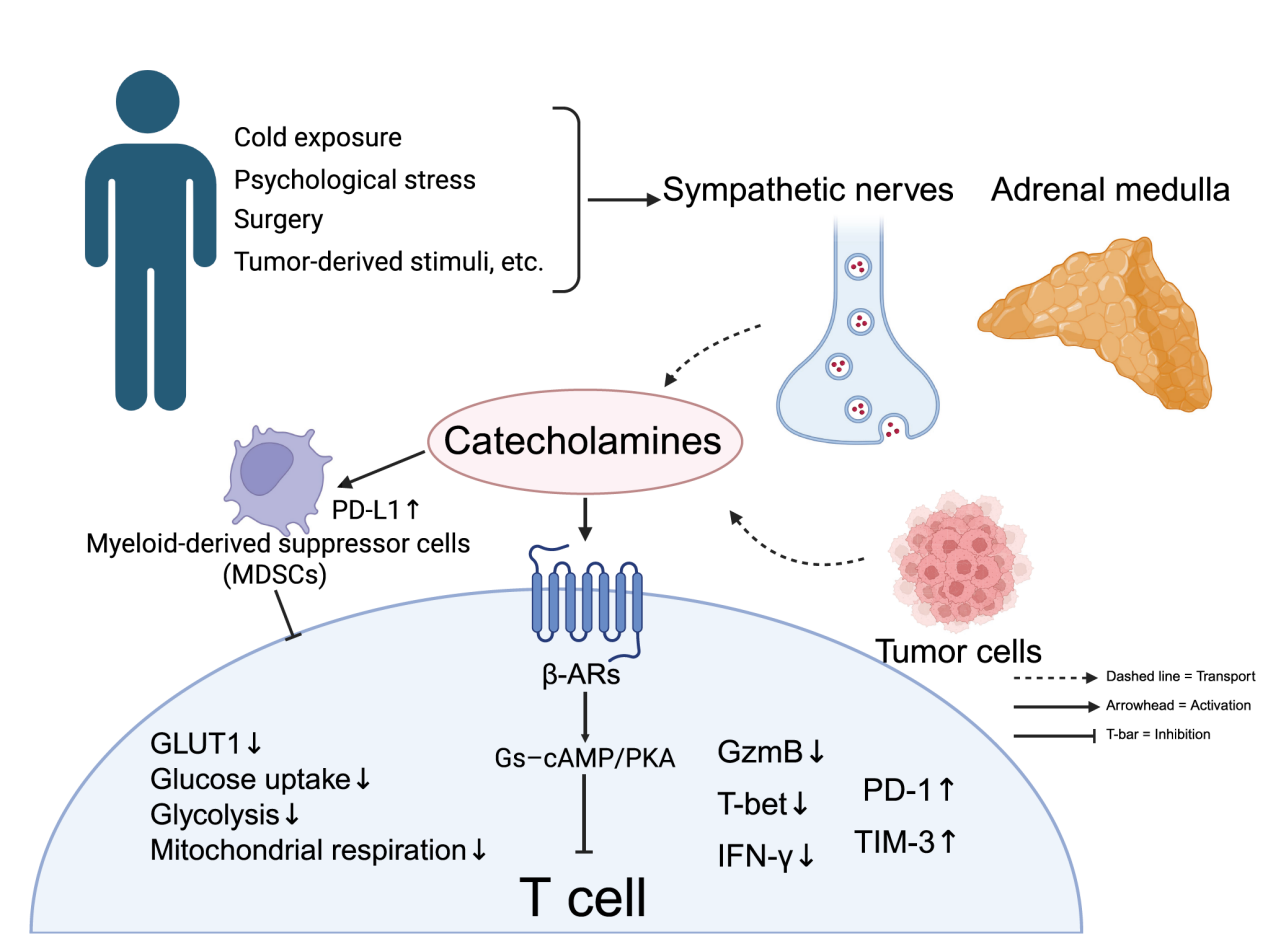
Figure 6 Adrenergic signaling in the tumor microenvironment suppresses T-cell metabolism and effector function Stress, cold exposure, surgery, and tumor-derived cues activate sympathetic nerves and the adrenal medulla, releasing catecholamines. In some tumors, cancer cells may also contribute to local catecholamine levels. These mediators act on the cAMP-PKA pathway, which reduces glucose uptake, glycolysis, mitochondrial respiration, and the expressions of effector molecules (granzyme B, T-bet, and IFN-γ while increasing the expression of inhibitory receptors (PD-1 and TIM-3). Catecholamines also enhance PD-L1 expression on myeloid-derived suppressor cells, further inhibiting T-cell activity.

Overall, the EPI/NE-β-adrenergic axis serves as a key link between neuroendocrine stress and T-cell immunity [165]. Its impact on cancer immunotherapy is highly context-dependent: chronic, high-level activation generally suppresses CTL responses and diminishes the efficacy of ICIs, CAR-T-cell therapy, and radiotherapy, whereas low-dose, acute activation can mobilize T cells [ 152, 166] . Prospectively designed, stratified clinical studies are urgently needed to define the benefits and risks of β-AR modulation across different stress patterns and transmitter levels and to identify and refine combination strategies that integrate β-blockade with immunotherapies to maximize patient benefit [ 140, 143, 167] ( Figure 6).

## New Technologies

In recent years, rapid advances in multi-omics and neuroscience tools have transformed approaches to studying neuro–immune interactions. Spatial transcriptomics (ST) and its multi-omics extensions now enable analysis of gene expression landscapes across cell populations with spatial resolution within tissues [ 168– 170] . In the tumor immune microenvironment, spatial multi-omics can finely map the proximity of nerve fibers to immune cells and reveal spatial associations between specific neurotransmitter signaling axes and T-cell functional states. For example, combining ST with immunohistochemistry can identify, at single-cell resolution, the distribution patterns of T-cell subsets expressing glutamate or adrenergic receptors within tumor-infiltrated regions [ 171– 174] .

Optogenetics and chemogenetics provide powerful tools to interrogate causality. By expressing light-gated ion channels ( *e*. *g*., ChR2) in defined neuronal populations or deploying designer receptors exclusively activated by designer drugs (DREADDs), investigators can selectively activate or inhibit neural circuits and then assess the impact on T-cell recruitment, activation, and effector function [ 175– 177] . These approaches have been validated in infectious and inflammatory models and show strong promise for applications in tumor immunology.

Neurotracing and denervation models can dissect the contribution of neural inputs to the tumor immune microenvironment. Anterograde and retrograde tracers label the types of nerve fibers projecting into tumor tissue; combined with surgical transection or chemical blockade of specific neural signals, these methods directly evaluate how loss of neural input affects T-cell infiltration and function. For example, peripheral sympathetic denervation can markedly alter the polarization of tumor-associated macrophages and the effector capacity of CD8 ^+^ T cells [178].

Single-cell RNA sequencing (scRNA-seq) integrated with T-cell receptor (TCR) sequencing offers an opportunity to link neurotransmitter receptor expression with T-cell clonal expansion [179]. Associating TCR sequences with the gene expression profiles of neurotransmitter receptors can reveal the clonal dynamics of T-cell subsets bearing specific receptor signatures during anti-tumor immunity. By linking TCR clonotypes with the transcriptional signatures of receptors such as GABBR1/2, HTR2A, or CHRM3, these datasets can reveal how neuromodulatory pathways influence T-cell expansion, exhaustion, and effector differentiation. The integration of single-cell multi-omics methods, such as ATAC-seq for chromatin accessibility and CITE-seq for surface proteins, further refines the regulatory context connecting neurotransmitter signaling with immunometabolic reprogramming [180]. Moreover, the integration of single-cell multi-omics (transcriptome + ATAC-seq + surface proteome) with spatial information may enable the reconstruction of neuro–immune microdomains and elucidate how distinct transmitter signals regulate immunometabolism, polarization, and trafficking [ 173, 181, 182] . These integrative maps delineate how receptor-expressing immune subsets colocalize with neural fibers or glial niches and how neurotransmitter gradients shape immune-cell states. For example, Slide-TCR-seq and SPTCR-seq achieve spatially resolved TCR and transcriptome co-profiling, revealing clonal positioning and local activation cues within tumor tissues [ 173, 183, 184] . Collectively, such integrative single-cell and spatial multi-omics frameworks provide a technical foundation for constructing high-resolution atlases of neural-immune interactions in the TME.

To refine the current pharmacological analysis framework, it is necessary to integrate multi-omics data mining approaches to systematically identify neurotransmitter-related therapeutic targets and predictive biomarkers. Through the combined use of spatial transcriptomics and single-cell sequencing, receptor expression patterns can be localized at cellular and tissue resolutions, providing a basis for mapping receptor heterogeneity within tumor and immune compartments. Subsequent receptor-ligand network inference enables the reconstruction of intercellular communication pathways that define neurotransmitter-mediated modulation of immune responses. Integrating metabolic flux data with receptor signaling analyses further elucidates the functional coupling between neurotransmitter metabolism and immune cell activation. These molecular and cellular insights can then be linked to clinical datasets and drug-repurposing resources to assess translational relevance and therapeutic feasibility. Quantitative assessment of druggability, structural accessibility, and pharmacokinetic suitability allows prioritization of viable molecular targets, whereas the integration of transcriptomic, proteomic, and metabolomic correlates supports the development of composite biomarker panels for patient stratification and efficacy prediction. This integrative analytical paradigm provides a robust, data-driven foundation for rational target discovery and precision drug repurposing, aligning with the recent multi-omics and network pharmacology frameworks proposed in Current Pharmaceutical Analysis [ 185– 187] .

## Microbiome-neurotransmitter Axis and Tumor Immunity

Recent evidence indicates that microbial communities within the host play pivotal roles in shaping tumor immunity through bidirectional interactions with the nervous and immune systems. The microbiome-neurotransmitter axis functions as a critical communication network linking microbial metabolism to neurotransmitter signaling pathways, thereby influencing immune regulation within the TME. Commensal and intratumoral bacteria can synthesize or metabolize key neurotransmitters such as acetylcholine (ACh), γ-aminobutyric acid (GABA), serotonin (5-HT), and catecholamines or can modulate their precursors and degradation products. For example, short-chain fatty acids (SCFAs) derived from microbial fermentation of dietary fibers upregulate host enzymes such as choline acetyltransferase (ChAT) and tryptophan hydroxylase-1 (TPH1), thereby enhancing cholinergic and serotonergic tone in immune cells [ 188, 189] . Microbial GABA production through glutamate decarboxylase (GAD) activity can alter dendritic cell differentiation and T-cell effector polarization, whereas dysbiosis characterized by reduced microbial diversity has been associated with lower intratumoral neurotransmitter availability, impaired antigen presentation, and resistance to immune checkpoint blockade [190]. These findings highlight that neurotransmitter flux in the tumor milieu is not solely neuron-derived but also modulated by microbial metabolism, which in turn establishes local immune thresholds and determines the amplitude of anti-tumor responses [ 191, 192] .

At the molecular and pharmacological levels, the microbiome–neurotransmitter axis exerts complex regulatory effects on receptor-mediated signaling and immune checkpoints. Microbial metabolites interact with neurotransmitter receptors expressed on tumor and immune cells, modulating canonical pathways such as GPCR-RTK coupling, cAMP/PKA-NF-κB signaling, and β-catenin-dependent chemokine repression [ 190, 193] . For instance, bacterial GABA can activate GABA_B receptors to suppress CCL4/CCL5 secretion and inhibit CD8 ^+^ T-cell recruitment, whereas microbial modulation of serotonin availability influences SERT-dependent uptake and T-cell metabolic activity [ 188, 189] . The restoration of microbial diversity through fecal microbiota transplantation (FMT) or probiotic administration has been shown to normalize neurotransmitter homeostasis, enhance dendritic-cell antigen presentation, and improve responsiveness to PD-1 blockade [194]. Integrative spatial transcriptomics combined with microbial detection (FISH/ISH or spatial meta-omics) provides a framework to map the co-localization of microbes, neurotransmitter synthesis or transport genes ( *e*. *g*., SLC6A4/SERT, CHRM1–3, GABBR1/2, and ADRB2), and immune-cell transcriptional states within the same tissue domain [188]. Collectively, these findings define the microbiome-neurotransmitter axis as an essential regulatory layer of tumor immunology, emphasizing that the coordinated analysis of microbial composition, neurotransmitter metabolism, and receptor signaling is crucial for predicting therapeutic response and designing precision interventions targeting tumor immunity.

## Membrane and Secreted Proteins in Neurotransmitter Regulation

Membrane proteins and secreted peptides serve as essential mediators in neurotransmitter-regulated immune communication, bridging neural signaling with immune modulation. Among them, ligand-gated ion channels and G protein-coupled receptors (GPCRs) act as the primary molecular conduits for neurotransmitter signals [ 9, 120, 195] . Ionotropic receptors such as NMDA, AMPA, and GABA_A directly regulate calcium influx and the membrane potential, thereby influencing immune-cell activation thresholds and cytokine secretion. In contrast, metabotropic receptors, including mGluR4, GABA_B, and 5-HT2A/5-HT7, couple to intracellular cascades such as MAPK, mTOR, and cAMP-PKA, modulating immune metabolism, antigen presentation, and the differentiation of T-cell subsets [ 196– 198] . These membrane proteins not only transmit neurotransmitter cues but also undergo ligand-dependent trafficking, internalization, and post-translational modification, which dynamically reshape receptor availability and signaling strength. Such fine-tuned regulation enables neurons and immune cells to coordinate their activities across inflammatory, degenerative, and tumor microenvironments.

Secreted proteins and peptides further amplify this neuro-immune crosstalk. Neuropeptides such as substance P, vasoactive intestinal peptide (VIP), neuropeptide Y (NPY), and calcitonin gene-related peptide (CGRP) bind to specific GPCRs on immune and glial cells, modulating the release of IL-10, IFN-γ, and TNF-α and thereby influencing the balance between pro- and anti-inflammatory states [ 77, 120, 196] . Secreted signaling proteins, including cytokine-like neurotrophic factors, can reciprocally regulate neurotransmitter receptor expression and membrane protein phosphorylation, reinforcing bidirectional communication between the nervous and immune systems [9]. Collectively, membrane and secreted proteins function as integrated signaling modules that synchronize neurotransmitter dynamics with immune surveillance and tolerance.

Given their structural diversity and high receptor selectivity, peptide drugs are emerging as promising modulators of neurotransmitter receptors in neural-immune regulation. Structure-guided and peptide engineering strategies have enabled the development of selective ligands targeting mGluR, GABA_B, and 5-HT receptor subtypes with improved stability and minimal off-target activity [199]. These agents can fine-tune immune signaling by promoting anti-inflammatory pathways or inhibiting excessive neuronal excitation. For instance, mGluR4 antagonistic peptides may restore dendritic cell activation and cytotoxic T-cell function, whereas peptidic GABA_B agonists can suppress neuroinflammation through the cAMP–CREB axis [ 24, 198] . Future development of these therapeutics should emphasize biased agonism to preferentially engage beneficial signaling routes, peripheral restriction to avoid central nervous system side effects, and nanocarrier delivery to achieve tissue-specific targeting. The integration of spatial transcriptomics and proteomics data will further support the rational design of peptide modulators tailored to patient-specific receptor landscapes. Together, these advances position peptide-based neurotransmitter modulators as a new class of precision neuroimmune therapeutics, offering novel avenues for treating immune dysfunction, neurodegeneration, and tumor progression.

## Conclusions

Overall, recent studies have demonstrated that neurotransmitters influence T cells and overall immune responses through multiple layers and mechanisms.

At the molecular level, different neurotransmitters and their receptors (such as NMDA receptors, mAChRs, β-adrenergic receptors, 5-HT receptors, and GABA_A/B receptors) directly regulate T-cell signaling pathways, including Ca² ^+^-dependent signaling, the cAMP-PKA pathway, MAPK cascades, and the mTOR metabolic axis. These signaling modulations not only reshape cytokine secretion profiles ( *e*. *g*., IFN-γ, IL-2, and IL-10) but also directly affect the cell cycle and survival programs.

At the cellular level, neurotransmitters modulate T-cell differentiation and effector states. For example, adrenergic signaling via β _2_ receptors suppresses Th1 immunity while promoting Th2 and Treg expansion, thereby creating an immunosuppressive bias. GABA signaling may inhibit Ca² ^+^ mobilization, thereby reducing CD8 ^+^ T-cell cytotoxicity. Conversely, glutamate-metabolism coupling can enhance T-cell activation and strengthen anti-tumor responses.

At the tissue microenvironment level, neurotransmitters contribute to the formation of so-called neuro-immune cell units (NICUs), which are functional microdomains composed of neurons, immune cells, and stromal elements. Within these units, neural terminals release neurotransmitters that act on neighboring T cells and antigen-presenting cells to fine-tune their functional states, thereby achieving precise regulation of immune responses.

At the systemic level, tumors can exploit neural plasticity to remodel local nerve density and neurotransmitter release patterns, reshaping the immune microenvironment. For example, certain tumors induce sympathetic remodeling, increasing norepinephrine release and thereby suppressing CD8 ^+^ T-cell effector function. This bidirectional interaction highlights neurotransmitters as not only modulators of immunity but also key participants in tumor immune evasion.

Overall, the multi-layered effects of neurotransmitters on T-cell immunity and tumor immune evasion can be systematically summarized. Table 1 provides an overview of the receptor-mediated signaling mechanisms and immunomodulatory consequences across major neurotransmitter classes, while Table 2 highlights neurotransmitter-linked pathways that contribute to immune checkpoint inhibitor (ICI) resistance and outlines rational combination strategies.

**Table:** **Table 1** Mechanisms and targeting strategies of neurotransmitter–receptor pathways in T cell immunomodulation

Neurotrans-mitter class	Receptors & T cell mechanisms	Net effect on T cell immunity	Targeting strategies (examples & combinations)	Ref.
Glutamate (Glu)	iGluR (AMPAR/NMDAR); mGluR1/4/5; TCR-coupled Lck/Akt and Ca²⁺; DC-released Glu tunes mGluR5→mGluR1; GRM4 restrains DC maturation; tumor/myeloid xCT exports Glu.	Context-dependent: generally co-stimulatory; GRM4 axis and high extracellular Glu can suppress.	Inhibit GRM4; modulate NMDAR/AMPAR ( *e.g*., perampanel); block or immunotarget xCT; reprogram glutamine-glutamate metabolism (JHU-083, CB-839); combine with PD-1/PD-L1 and TAM reprogramming.	[ 21– 24, 27, 33]
Acetylcholine (ACh)	nAChR (α7/α5/α9): JAK2/STAT3→↑PD‑L1; mAChR (M1–M5): PI3K-AKT, MAPK-ERK, Ca²/PKC; M3R→EGFR; ChAT⁺ CD4⁺ T cells restrain Ca²-NFAT; nicotine drives CD8⁺ exhaustion.	Bidirectional; exogenous nicotine generally impairs CD8⁺ effector function.	Block M3R ( *e.g*., darifenacin) or consider local denervation in select settings; avoid nicotine; investigational α7‑agonism (tumor-cell risk); transcuta-neous vagus stimulation as adjunct; combine with ICIs/radiotherapy.	[ 72– 76]
GABA	GABA_A (Cl⁻ channel; α3/GABRP subunits) ↓glycolysis and IFN‑γ; GABA_B (Gi/o) ↓cAMP/PKA, β‑cateninstabilization, ↓CCL4/5 → fewer CD8⁺/CD103⁺ DCs; ABAT/GAD1 set GABA burden; PD-L1 stabilization.	Broadly suppresses CD4⁺/CD8⁺ proliferation and effector function; magnitude depends on glucose/insulin, sex, and tumor type.	Reduce source (↓GAD1, ↑ABAT); GABA_B antagonists; subunit-selective GABA_A modulation ( *e.g*., target GABRP/α3); consider benzodiazepine‑sensitization of tumor cells with RT + anti-PD-L1; combine with PD-1/PD-L1; sequence/dose carefully.	[ 105, 107, 115– 117]
Serotonin (5-HT)	5-HT1A/1B (Gi), 5-HT2A/2B (Gq), 5-HT7 (Gs); TGM2‑mediated GAPDH serotonylation ↑glycolysis; MAO‑A limits T cell 5‑HT and polarizes TAMs; SERT (SLC6A4) clears intratumoral 5-HT.	Peripheral 5-HT tends to suppress immunity; intratumoral 5-HT can augment CD8⁺ effector function; receptor subtype and location matter.	Lower peripheral 5-HT (TPH1 inhibition); raise intratumoral 5-HT (SSRIs/SERTblock); MAO‑A inhibitors to reprogram TAMs; block 5-HT2A on T cells; 5-nonyloxytryp-tamine/tegaserod to ↑tumor MHC‑I; combine with PD-1/PD-L1 ± anti-VEGF.	[ 121, 122, 124, 127, 129– 131]
Catecholamines (NE/EPI)	β2‑AR→Gs/cAMP–PKA: ↓GLUT1, glycolysis, respiration; ↓T-bet/IFN-γ/GzmB; ↑PD‑1; expands MDSCs; α2‑AR autoreceptors ↓NE release; acute low‑dose EPI (exercise) can mobilize T cells.	Chronic/high-level β-adrenergic signaling suppresses CTL function and limits ICIs/RT; acute/low‑level EPI may transiently enhance immunity.	Non-selective β-blockade that includes β2-AR ( *e.g*., propranolol); α2‑AR agonists to limit NE; perioperative β‑blockade + COX‑2 inhibitor; ADRB2‑deleted CAR‑T; combine with PD-1/CTLA-4 and radiotherapy.	[ 145, 148, 151, 152, 157]

Notes: Effects are context‑dependent (receptor repertoire, timing/dose, tissue localization, metabolic state). Symbols: ↑ increase; ↓ decrease; → leads to.

Abbreviations: AMPAR, α-amino-3-hydroxy-5-methyl-4-isoxazolepropionic acid receptor; NMDAR, N-methyl-D-aspartate receptor; DC, dendritic cell; TIL, tumor-infiltrating lymphocyte; ICIs, immune checkpoint inhibitors; RT, radiotherapy; MAO-A, monoamine oxidase A; SERT, serotonin transporter; TPH1, tryptophan hydroxylase 1; xCT, SLC7A11; GOT1, glutamate-oxaloacetate transaminase 1; MDSC, myeloid-derived suppressor cell; ADRB2, β2-adrenergic receptor; EGFR, epidermal growth factor receptor.

**Table:** **Table 2** Neurotransmitter-linked mechanisms driving immune checkpoint inhibitor (ICI) resistance and potential combinatorial strategies

Neurotransmitter system	Key nodes	Mechanism	Impact on T cells / checkpoint axis	Ref.
Glutamate (Glu)	xCT/SLC7A11 (tumor, myeloid)	Glutamate efflux elevates extracellular Glu, rewires receptor signaling, increases Treg; anti-VEGF upregulates xCT; excess Glu impairs neutrophil cytotoxicity.	CD8⁺ infiltration/function↓; Treg↑; reduced responsiveness to PD-1/PD-L1 blockade.	[ 22, 23, 34– 38, 43]
GRM4 (mGluR4) on myeloid/DCs	GRM4 constrains DC maturation and antigen presentation, promoting a non-inflamed phenotype.	Impaired priming and CD8⁺/NK recruitment; suboptimal ICI responses.	[ 21, 24, 26]
NMDAR on TAMs; neuronal-tumor AMPAR synapses	NMDAR activation skews TAMs toward immunosuppression; AMPAR-mediated neuron-tumor synapses reinforce tumor excitability and immune evasion.	Myeloid niche reprogramming supports CD8⁺ activity when NMDAR is blocked; potential sensitization to PD-1 blockade.	[ 36, 44]
GOT1 (CD8⁺ T cell metabolism)	Loss of GOT1 diverts glutamate flux away from effector differentiation, limiting acute cytotoxic programs.	Effector programming and cytokines↓; weaker ICI benefit.	[ 26]
Acetylcholine (ACh)	α7-nAChR (tumor)	α7-nAChR→JAK2/STAT3 increases PD-L1 transcription and promotes immune escape under cholinergic input.	Tumor PD-L1↑; increased dependence on PD-1/PD-L1 axis.	[ 47, 96]
α9-nAChR (melanoma, others)	Nicotine→α9-nAChR engages AKT/ERK and STAT3 cross-talk, inducing PD-L1 and pro-migratory programs.	Sustained PD-L1; impaired CD8⁺ cytotoxicity.	[ 69]
α5-nAChR (lung adenocarcinoma)	α5-nAChR activates a STAT3-Jab1-PD-L1 axis integrating transcriptional induction with inhibition of PD-L1 ubiquitin‑mediated turnover.	Stabilized, elevated tumor PD-L1.	[ 68]
Exogenous nicotine/e-cig exposure→ T cell nAChRs	Remodels miRNA and elevates PD-1/CTLA-4 on T cells, reinforcing exhaustion and metastatic risk.	Exhausted CD8⁺ phenotypes; reduced ICI benefit.	[ 67, 83, 86]
M3 muscarinic receptor (M3R) (gastric/colorectal tumors)	ACh→M3R→EGFR/MAPK signaling promotes tumor growth and immunosuppressive milieu; receptor upregulation often coincides with PD-L1/PD-L2.	Proposed: increased checkpoint ligand expression and immune exclusion limit ICI efficacy.	[ 46, 47, 89, 90]
GABA	GABA_B receptor (tumor/myeloid)	GABA_B→β‑catenin lowers CCL4/CCL5, reduces CD8⁺ and CD103⁺ DC recruitment; promotes suppressive myeloid programs.	Immune exclusion; attenuated PD-1/PD-L1 responses.	[ 107, 114, 117]
B-cell-derived GABA→ IL-10⁺ macrophages	Activated B cells synthesize GABA that polarizes IL-10⁺ macrophages, suppressing CD8⁺ effector function.	Myeloid-mediated suppression of CD8⁺ cytotoxicity; ICI dampening.	[ 108]
ABAT loss (GABA catabolism)	ABAT downregulation increases GABA burden, favoring Treg differentiation and constraining CD8⁺ programs.	Treg↑; CD8⁺ effector molecules↓; weakened ICI responses.	[ 109, 112]
GABA_A α3 on CD8⁺; tumor-derived 4-Ac-GABA	4-Ac-GABA engages GABA_Aα3 to suppress AKT1 phosphorylation, lowering IFN-γ and granzyme B independent of TCR.	Exhaustion-like program; reduced cytotoxicity and ICI benefit.	[ 110]
GABA/baclofen→ PD-L1 stabilization	Downregulation of STUB1 reduces PD-L1 ubiquitination and stabilizes PD-L1 protein.	Tumor PD-L1↑; potential shift to anti-PD-L1 sensitivity.	[ 117, 118]
Serotonin (5-HT)	Peripheral 5-HT (platelet-stored)	Elevated systemic 5-HT dampens antitumor immunity; lowering peripheral 5-HT enhances CD8⁺ infiltration and potentiates PD-1/PD-L1 blockade.	Systemic effector attenuation; reversal upon 5-HT reduction.	[ 126, 128]
SERT on CD8⁺ T cells	SERT clears intratumoral 5-HT and limits autocrine activation; SSRIs inhibit SERT, increase local 5-HT, and augment ICI efficacy.	Enhanced CD8⁺ effector function; improved PD-1 responses when SERT is blocked.	[ 127]
Serotonin (5-HT)	5-HT1A on tumor cells	5-HT1A activation triggers autophagy/STAT3→PD‑L1 upregulation and Treg expansion; linked to poor outcomes in depression-associated LUAD.	Tumor PD‑L1↑; Tregs↑; reduced ICI efficacy.	[ 130– 132]
MAO-A (macrophages/T cells)	MAO-A drives suppressive TAM programs; inhibition reprograms TAMs and synergizes with PD-1 blockade.	Reduced suppressive TAM activity; improved T cell function.	[ 125– 133]
5-HT2A on T cells	Blocking 5-HT2A enhances CD8⁺ cytotoxic phenotype and tumor control; further benefit with anti-PD-L1 + anti-VEGF.	CD8⁺ activation↑; improved checkpoint responses.	[ 129]
Catecholamines (NE/EPI)	β2-AR on CD8⁺ TILs (chronic stress / cold)	β2‑AR→cAMP/PKA suppresses early metabolic reprogramming (GLUT1↓, glycolysis↓), T-bet/IFN-γ/GzmB↓ and PD-1↑; limits response to PD-1 blockade.	Terminal exhaustion features and low metabolic fitness in TILs.	[ 140, 142, 149]
NE→Wnt7A-β-catenin (LUAD)	NE activates Wnt7A-β-catenin, decreasing CXCL9 and increasing adenosine, limiting CD8⁺ recruitment and creating PD‑1 resistance.	Immune exclusion with low CD8⁺ infiltration.	[ 160]
β2-AR-conditioned MDSCs	β-adrenergic signaling sustains MDSC survival and suppressive function (ARG1↑, PD‑L1↑), constraining T cell responses.	Myeloid PD-L1↑; T cell suppression; poorer ICI outcomes.	[ 19, 152]
β3-AR on TILs (neuroblastoma)	β3-AR signaling maintains IFN-γ-driven tumor PD-L1 in a feedback loop that limits T cell cytotoxicity.	Sustained tumor PD-L1; reduced efficacy of ICIs.	[ 159]
α2-AR activation (presynaptic)	α2-AR agonism lowers NE release, indirectly mitigating β2-AR-driven suppression; increases TIL infiltration and improves anti-PD-1 responses.	Restored CTL trafficking and effector programs.	[ 147, 152]

Notes: Effects are context-dependent (receptor repertoire, timing/dose, tissue localization, metabolic state). Mechanisms and strategies summarized from refs. listed in the table and corresponding main-text sections. Symbols: ↑ increase; ↓ decrease; → leads to.

Abbreviations: AMPAR, α-amino-3-hydroxy-5-methyl-4-isoxazolepropionic acid receptor; DC, dendritic cell; GBM, glioblastoma; GC, gastric cancer; CRC, colorectal cancer; HCC, hepatocellular carcinoma; ICI, immune checkpoint inhibitor; LUAD, lung adenocarcinoma; MAO-A, monoamine oxidase A; MDSC, myeloid-derived suppressor cell; NE, norepinephrine; EPI, epinephrine; PD-1, programmed cell death protein 1; PD-L1, programmed death ligand 1; TAM, tumor-associated macrophage; TIL, tumor-infiltrating lymphocyte; Treg, regulatory T cell.

In summary, the integrated application of spatial multi-omics, optogenetics and chemogenetics, neurotracing and denervation models, and single-cell immune sequencing can reveal, at spatial, functional, and dynamic levels, the full landscape of neurotransmitter–receptor regulation of T-cell immunity. Peptide ligands and neuropeptides add a crucial dimension to neuro–immune regulation. Their receptor-selective modulation may offer new avenues for precise and context-specific immunotherapy. These insights provide a solid foundation for future tumor immunotherapy strategies that target neuro-immune interactions.

## References

[1] Kumar BV, Connors TJ, Farber DL (2018). Human T cell development, localization, and function throughout life. Immunity.

[2] Zou D, Li XC, Chen W (2025). Beyond T-cell subsets: stemness and adaptation redefining immunity and immunotherapy. Cell Mol Immunol.

[3] Farhood B, Najafi M, Mortezaee K (2019). CD8
^+^ cytotoxic T lymphocytes in cancer immunotherapy: a review. J Cell Physiol.

[4] Zhu J, Paul WE (2010). Heterogeneity and plasticity of T helper cells. Cell Res.

[5] Luckheeram RV, Zhou R, Verma AD, Xia B (2012). CD4
^+^ T cells: differentiation and functions. Clin Dev Immunol.

[6] Sharma P, Allison JP (2015). The future of immune checkpoint therapy. Science.

[7] Togashi Y, Shitara K, Nishikawa H (2019). Regulatory T cells in cancer immunosuppression-implications for anticancer therapy. Nat Rev Clin Oncol.

[8] Elenkov IJ, Wilder RL, Chrousos GP, Vizi ES (2000). The sympathetic nerve-an integrative interface between two supersystems: the brain and the immune system. Pharmacol Rev.

[9] Pavlov VA, Tracey KJ (2017). Neural regulation of immunity: molecular mechanisms and clinical translation. Nat Neurosci.

[10] Godinho-Silva C, Cardoso F, Veiga-Fernandes H (2019). Neuro–immune cell units: a new paradigm in physiology. Annu Rev Immunol.

[11] Kawashima K (2004). Expression of non-neuronal acetylcholine in lymphocytes and its contribution to the regulation of immune function. Front Biosci.

[12] Cole SW, Sood AK (2012). Molecular pathways: beta-adrenergic signaling in cancer. Clin Cancer Res.

[13] Kobrzycka A, Napora P, Pearson BL, Pierzchała-Koziec K, Szewczyk R, Wieczorek M (2019). Peripheral and central compensatory mechanisms for impaired vagus nerve function during peripheral immune activation. J Neuroinflamm.

[14] Tracey KJ (2002). The inflammatory reflex. Nature.

[15] Besedovsky HO, del Rey A (2011). Central and peripheral cytokines mediate immune-brain connectivity. Neurochem Res.

[16] Zahalka AH, Frenette PS (2020). Nerves in cancer. Nat Rev Cancer.

[17] Mauffrey P, Tchitchek N, Barroca V, Bemelmans AP, Firlej V, Allory Y, Roméo PH (2019). Progenitors from the central nervous system drive neurogenesis in cancer. Nature.

[18] Gysler SM, Drapkin R (2021). Tumor innervation: peripheral nerves take control of the tumor microenvironment. J Clin Invest.

[19] Mohammadpour H, MacDonald CR, Qiao G, Chen M, Dong B, Hylander BL, McCarthy PL (2019). β2 adrenergic receptor–mediated signaling regulates the immunosuppressive potential of myeloid-derived suppressor cells. J Clin Invest.

[20] Minogue E, Cunha PP, Wadsworth BJ, Grice GL, Sah-Teli SK, Hughes R, Bargiela D (2023). Glutarate regulates T cell metabolism and anti-tumour immunity. Nat Metab.

[21] Wan Z, Sun R, Liu YW, Li S, Sun J, Li J, Zhu J (2021). Targeting metabotropic glutamate receptor 4 for cancer immunotherapy. Sci Adv.

[22] Lim JKM, Delaidelli A, Minaker SW, Zhang HF, Colovic M, Yang H, Negri GL (2019). Cystine/glutamate antiporter xCT (SLC7A11) facilitates oncogenic RAS transformation by preserving intracellular redox balance. Proc Natl Acad Sci USA.

[23] Koppula P, Zhang Y, Shi J, Li W, Gan B (2017). The glutamate/cystine antiporter SLC7A11/xCT enhances cancer cell dependency on glucose by exporting glutamate. J Biol Chem.

[24] Ju X, Putz EM, Liu Y, Yuan D, Sun G, Koda S, Fu Z (2025). Metabotropic glutamate receptor 4-mediated glutamatergic signaling reshapes the tumor microenvironment by regulating dendritic cell maturation. Nat Commun.

[25] Pacheco R, Oliva H, Martinez-Navío JM, Climent N, Ciruela F, Gatell JM, Gallart T (2006). Glutamate released by dendritic cells as a novel modulator of T cell activation. J Immunol.

[26] Xu W, Patel CH, Zhao L, Sun IH, Oh MH, Sun IM, Helms RS (2023). GOT1 regulates CD8+ effector and memory T cell generation. Cell Rep.

[27] Koda S, Hu J, Ju X, Sun G, Shao S, Tang RX, Zheng KY (2023). The role of glutamate receptors in the regulation of the tumor microenvironment. Front Immunol.

[28] Mairhofer DG, Ortner D, Tripp CH, Schaffenrath S, Fleming V, Heger L, Komenda K (2015). Impaired gp100-Specific CD8+ T-cell responses in the presence of myeloid-derived suppressor cells in a spontaneous mouse melanoma model. J Investig Dermatol.

[29] Ganor Y, Besser M, Ben-Zakay N, Unger T, Levite M (2003). Human T cells express a functional ionotropic glutamate receptor glur3, and glutamate by itself triggers integrin-mediated adhesion to laminin and fibronectin and chemotactic migration. J Immunol.

[30] Ganor Y, Teichberg VI, Levite M (2007). TCR activation eliminates glutamate receptor glur3 from the cell surface of normal human T cells, via an autocrine/paracrine granzyme b-mediated proteolytic cleavage. J Immunol.

[31] Pacheco R, Ciruela F, Casadó V, Mallol J, Gallart T, Lluis C, Franco R (2004). Group I metabotropic glutamate receptors mediate a dual role of glutamate in T cell activation. J Biol Chem.

[32] Shanker A, de Aquino MTP, Hodo TW, Uzhachenko R (2020). Glutamate receptor signaling is critical for T cell function and antitumor activity. J Immunol.

[33] Shanker A, de Aquino MTP, Hodo T, Uzhachenko R (2018). Glutamate receptors provide costimulatory signals to improve T cell immune response. J Immunol.

[34] Varghese S, Pramanik S, Williams LJ, Hodges HR, Hudgens CW, Fischer GM, Luo CK (2021). The glutaminase inhibitor CB-839 (telaglenastat) enhances the antimelanoma activity of T-cell–mediated immunotherapies. Mol Cancer Ther.

[35] Leone RD, Zhao L, Englert JM, Sun IM, Oh MH, Sun IH, Arwood ML (2019). Glutamine blockade induces divergent metabolic programs to overcome tumor immune evasion. Science.

[36] Long Y, Tao H, Karachi A, Grippin AJ, Jin L, Chang YE, Zhang W (2020). Dysregulation of glutamate transport enhances treg function that promotes vegf blockade resistance in glioblastoma. Cancer Res.

[37] Tang B, Wang Y, Xu W, Zhu J, Weng Q, Chen W, Fang S (2023). Macrophage xCT deficiency drives immune activation and boosts responses to immune checkpoint blockade in lung cancer. Cancer Lett.

[38] Xiong T, He P, Zhou M, Zhong D, Yang T, He W, Xu Z (2022). Glutamate blunts cell-killing effects of neutrophils in tumor microenvironment. Cancer Sci.

[39] Wen Z, Liu T, Xu X, Acharya N, Shen Z, Lu Y, Xu J (2025). Interleukin-16 enhances anti-tumor immune responses by establishing a Th1 cell-macrophage crosstalk through reprogramming glutamine metabolism in mice. Nat Commun.

[40] Goldshmit Y, Perelroizen R, Yakovchuk A, Banyas E, Mayo L, David S, Benbenishty A (2021). Blood glutamate scavengers increase pro-apoptotic signaling and reduce metastatic melanoma growth
*in vivo*. Sci Rep.

[41] Karati D, Kumar D (2024). Molecular insight into the apoptotic mechanism of cancer cells: an explicative review. Curr Mol Pharmacol.

[42] Tobochnik S, Regan MS, Dorotan MKC, Reich D, Lapinskas E, Hossain MA, Stopka S (2024). Pilot trial of perampanel on peritumoral hyperexcitability in newly diagnosed high-grade glioma. Clin Cancer Res.

[43] Conti L, Bolli E, Di Lorenzo A, Franceschi V, Macchi F, Riccardo F, Ruiu R (2020). Immunotargeting of the xCT cystine/glutamate antiporter potentiates the efficacy of her2-targeted immunotherapies in breast cancer. Cancer Immunol Res.

[44] Yuan D, Hu J, Ju X, Putz EM, Zheng S, Koda S, Sun G (2023). NMDAR antagonists suppress tumor progression by regulating tumor-associated macrophages. Proc Natl Acad Sci USA.

[45] Wessler I, Kirkpatrick CJ (2008). Acetylcholine beyond neurons: the non-neuronal cholinergic system in humans. Br J Pharmacol.

[46] Kawashima K (2000). Extraneuronal cholinergic system in lymphocytes. Pharmacol Ther.

[47] Rosas-Ballina M, Olofsson PS, Ochani M, Valdés-Ferrer SI, Levine YA, Reardon C, Tusche MW (2011). Acetylcholine-synthesizing T cells relay neural signals in a vagus nerve circuit. Science.

[48] Fujii T, Mashimo M, Moriwaki Y, Misawa H, Ono S, Horiguchi K, Kawashima K (2017). Physiological functions of the cholinergic system in immune cells. J Pharmacol Sci.

[49] Ferguson SM, Bazalakova M, Savchenko V, Tapia JC, Wright J, Blakely RD (2004). Lethal impairment of cholinergic neurotransmission in hemicholinium-3-sensitive choline transporter knockout mice. Proc Natl Acad Sci USA.

[50] Parsons SM (2000). Transport mechanisms in acetylcholine and monoamine storage. FASEB.

[51] Lips KS, Volk C, Schmitt BM, Pfeil U, Arndt P, Miska D, Ermert L,
*et al*. Polyspecific cation transporters mediate luminal release of acetylcholine from bronchial epithelium.
*
Am J Respir Cell Mol Biol
* 2005, 33: 79–88. https://doi.org/10.1165/rcmb.2004-0363°C.

[52] Kummer W, Lips KS, Pfeil U (2008). The epithelial cholinergic system of the airways. Histochem Cell Biol.

[53] Zheng C, Snow BE, Elia AJ, Nechanitzky R, Dominguez-Brauer C, Liu S, Tong Y (2023). Tumor-specific cholinergic CD4+ T lymphocytes guide immunosurveillance of hepatocellular carcinoma. Nat Cancer.

[54] Reijmen E, De Mey S, Van Damme H, De Ridder K, Gevaert T, De Blay E, Bouwens L (2021). Transcutaneous vagal nerve stimulation alone or in combination with radiotherapy stimulates lung tumor infiltrating lymphocytes but fails to suppress tumor growth. Front Immunol.

[55] Eil R, Vodnala SK, Clever D, Klebanoff CA, Sukumar M, Pan JH, Palmer DC (2016). Ionic immune suppression within the tumour microenvironment limits T cell effector function. Nature.

[56] Yu H, Xia H, Tang Q, Xu H, Wei G, Chen Y, Dai X (2017). Acetylcholine acts through M3 muscarinic receptor to activate the EGFR signaling and promotes gastric cancer cell proliferation. Sci Rep.

[57] Magnon C, Hall SJ, Lin J, Xue X, Gerber L, Freedland SJ, Frenette PS (2013). Autonomic nerve development contributes to prostate cancer progression. Science.

[58] Hernandez CA, Verzeroli C, Roca Suarez AA, Farca-Luna AJ, Tonon L, Esteban-Fabró R, Pinyol R (2025). Hepatocellular carcinoma hosts cholinergic neural cells and tumoral hepatocytes harboring targetable muscarinic receptors. JHEP Rep.

[59] Albuquerque EX, Pereira EFR, Alkondon M, Rogers SW (2009). Mammalian nicotinic acetylcholine receptors: from structure to function. Physiol Rev.

[60] Caulfield MP, Birdsall NJM (1998). International union of pharmacology. XVII. classification of muscarinic acetylcholine receptors. Pharmacol Rev.

[61] Dani JA, Bertrand D (2007). Nicotinic acetylcholine receptors and nicotinic cholinergic mechanisms of the central nervous system. Annu Rev Pharmacol Toxicol.

[62] Improgo MRD, Scofield MD, Tapper AR, Gardner PD (2010). From smoking to lung cancer: the CHRNA5/A3/B4 connection. Oncogene.

[63] Schuller HM (2009). Is cancer triggered by altered signalling of nicotinic acetylcholine receptors?. Nat Rev Cancer.

[64] Egleton RD, Brown KC, Dasgupta P (2008). Nicotinic acetylcholine receptors in cancer: multiple roles in proliferation and inhibition of apoptosis. Trends Pharmacol Sci.

[65] Wang N, Liang H, Zen K (2014). Molecular mechanisms that influence the macrophage M1-M2 polarization balance. Front Immunol.

[66] Wang GZ, Zhang L, Zhao XC, Gao SH, Qu LW, Yu H, Fang WF (2019). The Aryl hydrocarbon receptor mediates tobacco-induced PD-L1 expression and is associated with response to immunotherapy. Nat Commun.

[67] Cheng CC, Lin HC, Chiang YW, Chang J, Sie ZL, Yang BL, Lim KH (2021). Nicotine exhausts CD8+ T cells against tumor cells through increasing miR-629-5p to repress IL2RB-mediated granzyme B expression. Cancer Immunol Immunother.

[68] Zhu P, Jin Z, Kang G, Jia Y, Liu D, Zhang Q, Guo F (2022). Alpha5 nicotinic acetylcholine receptor mediated immune escape of lung adenocarcinoma via STAT3/Jab1-PD-L1 signalling. Cell Commun Signal.

[69] Nguyen HD, Liao YC, Ho YS, Chen LC, Chang HW, Cheng TC, Liu D (2019). The α9 nicotinic acetylcholine receptor mediates nicotine-induced PD-L1 expression and regulates melanoma cell proliferation and migration. Cancers.

[70] Wang J, Cai J, Wang Z, Yang S, Wang J, Jia Y, Sun H (2025). α5-nAChR/NETO2 contributed to chronic stress-promoted lung adenocarcinoma progression. Cancer Cell Int.

[71] Frullanti E, Galvan A, Falvella FS, Manenti G, Colombo F, Vannelli A, Incarbone M (2011). Multiple genetic loci modulate lung adenocarcinoma clinical staging. Clin Cancer Res.

[72] Kruse AC, Kobilka BK, Gautam D, Sexton PM, Christopoulos A, Wess J (2014). Muscarinic acetylcholine receptors: novel opportunities for drug development. Nat Rev Drug Discov.

[73] Thal DM, Sun B, Feng D, Nawaratne V, Leach K, Felder CC, Bures MG (2016). Crystal structures of the M1 and M4 muscarinic acetylcholine receptors. Nature.

[74] Zhao CM, Hayakawa Y, Kodama Y, Muthupalani S, Westphalen CB, Andersen GT, Flatberg A (2014). Denervation suppresses gastric tumorigenesis. Sci Transl Med.

[75] Renz BW, Tanaka T, Sunagawa M, Takahashi R, Jiang Z, Macchini M, Dantes Z (2018). Cholinergic signaling via muscarinic receptors directly and indirectly suppresses pancreatic tumorigenesis and cancer stemness. Cancer Discov.

[76] Saloman JL, Albers KM, Li D, Hartman DJ, Crawford HC, Muha EA, Rhim AD (2016). Ablation of sensory neurons in a genetic model of pancreatic ductal adenocarcinoma slows initiation and progression of cancer. Proc Natl Acad Sci USA.

[77] Manohar SM (2023). At the crossroads of TNF α signaling and cancer. Curr Mol Pharmacol.

[78] Fujii T, Mashimo M, Moriwaki Y, Misawa H, Ono S, Horiguchi K, Kawashima K (2017). Expression and function of the cholinergic system in immune cells. Front Immunol.

[79] Rosas‐Ballina M, Tracey KJ (2009). Cholinergic control of inflammation. J Internal Med.

[80] Andersson U, Tracey KJ (2012). Neural reflexes in inflammation and immunity. J Exp Med.

[81] Zhang J, Summah H, Zhu Y, Qu JM (2011). Nicotinic acetylcholine receptor variants associated with susceptibility to chronic obstructive pulmonary disease: a meta-analysis. Respir Res.

[82] Naeimzadeh Y, Heidari Z, Razban V, Khajeh S (2023). Deregulated microRNAs involved in P53 signaling pathway in breast cancer with focus on triple-negative breast cancer. Curr Mol Pharmacol.

[83] Wasén C, Ospelt C, Camponeschi A, Erlandsson MC, Andersson KME, Silfverswärd ST, Gay S (2020). Nicotine changes the microRNA profile to regulate the FOXO memory program of CD8+ T cells in rheumatoid arthritis. Front Immunol.

[84] Buck MD, O’Sullivan D, Pearce EL (2015). T cell metabolism drives immunity. J Exp Med.

[85] Keshavarzmotamed A, Mousavi V, Masihipour N, Rahmati A, Mousavi Dehmordi R, Ghezelbash B, Alimohammadi M (2023). Regulating miRNAs expression by resveratrol: novel insights based on molecular mechanism and strategies for cancer therapy. Curr Mol Pharmacol.

[86] Arias-Badia M, Pai CCS, Chen PX, Chang A, Lwin YM, Srinath A, Gotts JE (2024). E-cigarette exposure disrupts antitumor immunity and promotes metastasis. Front Immunol.

[87] Sakaguchi S, Yamaguchi T, Nomura T, Ono M (2008). Regulatory T cells and immune tolerance. Cell.

[88] Kuol N, Godlewski J, Kmiec Z, Vogrin S, Fraser S, Apostolopoulos V, Nurgali K (2023). Cholinergic signaling influences the expression of immune checkpoint inhibitors, PD-L1 and PD-L2, and tumor hallmarks in human colorectal cancer tissues and cell lines. BMC Cancer.

[89] Wang L, Xu J, Xia Y, Yin K, Li Z, Li B, Wang W (2018). Muscarinic acetylcholine receptor 3 mediates vagus nerve-induced gastric cancer. Oncogenesis.

[90] Wessler IK, Kirkpatrick CJ (2001). The non-neuronal cholinergic system: an emerging drug target in the airways. Pulmonary Pharmacol Ther.

[91] Watson GA, Sanz-Garcia E, Zhang WJ, Liu ZA, Yang SC, Wang B, Liu S (2022). Increase in serum choline levels predicts for improved progression-free survival (PFS) in patients with advanced cancers receiving pembrolizumab. J Immunother Cancer.

[92] Freedman R, Olincy A, Buchanan RW, Harris JG, Gold JM, Johnson L, Allensworth D (2008). Initial phase 2 trial of a nicotinic agonist in schizophrenia. Am J Psychiatry.

[93] Gattenlöhner S, Marx A, Markfort B, Pscherer S, Landmeier S, Juergens H, Müller-Hermelink HK (2006). Rhabdomyosarcoma lysis by T cells expressing a human autoantibody-based chimeric receptor targeting the fetal acetylcholine receptor. Cancer Res.

[94] Kramer K, Brannan SK, Sauder C, Kaul I (2025). Safety and efficacy of karxt in patients with schizophrenia in the randomized, double-blind, placebo-controlled emergent trials. Int J NeuropsychoPharmacol.

[95] Hering NA, Liu V, Kim R, Weixler B, Droeser RA, Arndt M, Pozios I (2021). Blockage of cholinergic signaling via muscarinic acetylcholine receptor 3 inhibits tumor growth in human colorectal adenocarcinoma. Cancers.

[96] Kox M, Pompe JC, Gordinou de Gouberville MC, van der Hoeven JG, Hoedemaekers CW, Pickkers P (2011). Effects of the α7 nicotinic acetylcholine receptor agonist Gts-21 on the innate immune response in humans. Shock.

[97] Schaal C, Chellappan SP (2014). Nicotine-mediated cell proliferation and tumor progression in smoking-related cancers. Mol Cancer Res.

[98] Rosas-Ballina M, Goldstein RS, Gallowitsch-Puerta M, Yang L, Valdés-Ferrer SI, Patel NB, Chavan S (2009). The selective α7 agonist GTS-21 attenuates cytokine production in human whole blood and human monocytes activated by ligands for TLR2, TLR3, TLR4, TLR9, and RAGE. Mol Med.

[99] Brannan SK, Sawchak S, Miller AC, Lieberman JA, Paul SM, Breier A (2021). Muscarinic cholinergic receptor agonist and peripheral antagonist for schizophrenia. N Engl J Med.

[100] Breier A, Brannan SK, Paul SM, Miller AC (2023). Evidence of trospium’s ability to mitigate cholinergic adverse events related to xanomeline: phase 1 study results. Psychopharmacology.

[101] Correll CU, Angelov AS, Miller AC, Weiden PJ, Brannan SK (2022). Safety and tolerability of KarXT (xanomeline–trospium) in a phase 2, randomized, double-blind, placebo-controlled study in patients with schizophrenia. Schizophrenia.

[102] Bhat R, Axtell R, Mitra A, Miranda M, Lock C, Tsien RW, Steinman L (2010). Inhibitory role for GABA in autoimmune inflammation. Proc Natl Acad Sci USA.

[103] Tian J, Chau C, Hales TG, Kaufman DL (1999). GABA(A) receptors mediate inhibition of T cell responses. J Neuroimmunol.

[104] Tian J, Lu Y, Zhang H, Chau CH, Dang HN, Kaufman DL (2004). γ-aminobutyric acid inhibits T cell autoimmunity and the development of inflammatory responses in a mouse type 1 diabetes model. J Immunol.

[105] Bhandage AK, Jin Z, Korol SV, Shen Q, Pei Y, Deng Q, Espes D (2018). GABA regulates release of inflammatory cytokines from peripheral blood mononuclear cells and CD4+ T cells and is immunosuppressive in type 1 diabetes. eBioMedicine.

[106] Bjurstöm H, Wang JY, Ericsson I, Bengtsson M, Liu Y, Kumar-Mendu S, Issazadeh-Navikas S (2008). GABA, a natural immunomodulator of T lymphocytes. J Neuroimmunol.

[107] Huang D, Wang Y, Thompson JW, Yin T, Alexander PB, Qin D, Mudgal P (2022). Cancer-cell-derived GABA promotes β-catenin-mediated tumour growth and immunosuppression. Nat Cell Biol.

[108] Zhang B, Vogelzang A, Miyajima M, Sugiura Y, Wu Y, Chamoto K, Nakano R (2021). B cell-derived GABA elicits IL-10+ macrophages to limit anti-tumour immunity. Nature.

[109] Kang S, Liu L, Wang T, Cannon M, Lin P, Fan TWM, Scott DA (2022). GAB functions as a bioenergetic and signalling gatekeeper to control T cell inflammation. Nat Metab.

[110] Zhou X, Chen Z, Yu Y, Li M, Cao Y, Prochownik EV, Li Y (2024). Increases in 4-acetaminobutyric acid generated by phosphomevalonate kinase suppress CD8
^+^ T cell activation and allow tumor immune escape. Adv Sci.

[111] Jiang SH, Zhu LL, Zhang M, Li RK, Yang Q, Yan JY, Zhang C (2019). GABRP regulates chemokine signalling, macrophage recruitment and tumour progression in pancreatic cancer through tuning KCNN4-mediated Ca
^2+^ signalling in a GABA-independent manner. Gut.

[112] Dong Y, Wang G, Nie D, Xu Y, Bai X, Lu C, Jian F (2024). Tumor-derived GABA promotes lung cancer progression by influencing TAMs polarization and neovascularization. Int Immunopharmacol.

[113] Han H, Lu Y, Xu S, Zhang W, Lin W, Zhan J, Chen G (2025). Overcoming GABA-induced treg suppression of immunity by ABAT to augment CD8
^+^ T cell antitumor immune response in liver cancer. Int Arch Allergy Immunol.

[114] Sun S, Chen X, Ding N, Zhang M, Li X, Chen L, Sun K (2024). Gamma-aminobutyric acid-mediated neuro-immune interactions in glioblastoma: implications for prognosis and immunotherapy response. Life Sci.

[115] Jin Z, Hammoud H, Bhandage AK, Korol SV, Trujeque-Ramos O, Koreli S, Gong Z (2024). GABA-mediated inhibition of human CD4+ T cell functions is enhanced by insulin but impaired by high glucose levels. eBioMedicine.

[116] Sparrow EL, James S, Hussain K, Beers SA, Cragg MS, Bogdanov YD, Obukhov AG (2021). Activation of GABA(A) receptors inhibits T cell proliferation. PLoS One.

[117] Sun X, Lin M, Tian Z, Ma Y, Lv L (2024). GABA/baclofen stabilizes PD-L1 and enhances immunotherapy of breast cancer. Heliyon.

[118] Zhang X, Zhang R, Zheng Y, Shen J, Xiao D, Li J, Shi X (2013). Expression of gamma-aminobutyric acid receptors on neoplastic growth and prediction of prognosis in non-small cell lung cancer. J Transl Med.

[119] Pomeranz Krummel DA, Nasti TH, Kaluzova M, Kallay L, Bhattacharya D, Melms JC, Izar B (2021). Melanoma cell intrinsic GABAA receptor enhancement potentiates radiation and immune checkpoint inhibitor response by promoting direct and T cell-mediated antitumor activity. Int J Radiat Oncol Biol Phys.

[120] Hodo TW, de Aquino MTP, Shimamoto A, Shanker A (2020). Critical neurotransmitters in the neuroimmune network. Front Immunol.

[121] Wu H, Herr DV, MacIver NJ, Rathmell JC, Gerriets VA (2020). CD4 T cells differentially express cellular machinery for serotonin signaling, synthesis, and metabolism. Int Immunopharmacol.

[122] León-Ponte, M, Ahern GP, O′Connell PJ (2007). Serotonin provides an accessory signal to enhance T-cell activation by signaling through the 5-HT7 receptor. Blood.

[123] Yin J, Albert RH, Tretiakova AP, Jameson BA (2006). 5-HT1B receptors play a prominent role in the proliferation of T-lymphocytes. J Neuroimmunol.

[124] Wang X, Fu SQ, Yuan X, Yu F, Ji Q, Tang HW, Li RK (2024). A GAPDH serotonylation system couples CD8+ T cell glycolytic metabolism to antitumor immunity. Mol Cell.

[125] Wang X, Li B, Kim YJ, Wang YC, Li Z, Yu J, Zeng S (2021). Targeting monoamine oxidase a for T cell–based cancer immunotherapy. Sci Immunol.

[126] Schneider MA, Heeb L, Beffinger MM, Pantelyushin S, Linecker M, Roth L, Lehmann K (2021). Attenuation of peripheral serotonin inhibits tumor growth and enhances immune checkpoint blockade therapy in murine tumor models. Sci Transl Med.

[127] Li B, Elsten-Brown J, Li M, Zhu E, Li Z, Chen Y, Kang E (2025). Serotonin transporter inhibits antitumor immunity through regulating the intratumoral serotonin axis. Cell.

[128] Yasen M, Sun N, Jia J, Hong W, Zhuang L, Huang J, Chen X (2025). Serotonin metabolism shapes the tumor immune microenvironment and serves as atherapeutic target in lung cancer. Anticancer Agents Med Chem.

[129] Tay RE, Ho CM, Ang NDZ, Tay HC, Lopez DZ, Na QR, Tan YW (2025). Serotonin receptor 5-HT
_2A_ as a potential target for HCC immunotherapy. J Immunother Cancer.

[130] Stachura P, Liu W, Xu HC, Wlodarczyk A, Stencel O, Pandey P, Vogt M (2023). Unleashing T cell anti-tumor immunity: new potential for 5-Nonloxytryptamine as an agent mediating MHC-I upregulation in tumors. Mol Cancer.

[131] Qin X, Li J, Wang S, Lv J, Luan F, Liu Y, Chen Y (2021). Serotonin/HTR1E signaling blocks chronic stress-promoted progression of ovarian cancer. Theranostics.

[132] Liu Y, Zhang H, Wang ZY, Wu P, Gong W (2019). 5-Hydroxytryptamine1a receptors on tumour cells induce immune evasion in lung adenocarcinoma patients with depression via autophagy/pSTAT3. Eur J Cancer.

[133] Wang YC, Wang X, Yu J, Ma F, Li Z, Zhou Y, Zeng S (2021). Targeting monoamine oxidase a-regulated tumor-associated macrophage polarization for cancer immunotherapy. Nat Commun.

[134] Brown J, Li Z, Wang X, Kim YJ, Wang YC, Zuo Y, Hong W (2022). Nanoformulation improves antitumor efficacy of MAOI immune checkpoint blockade therapy without causing aggression-related side effects. Front Pharmacol.

[135] Yang Z, Li Z, Guo Z, Ren Y, Zhou T, Xiao Z, Duan J (2021). Antitumor effect of fluoxetine on chronic stress-promoted lung cancer growth via suppressing kynurenine pathway and enhancing cellular immunity. Front Pharmacol.

[136] Dong F, He K, Zhang S, Song K, Jiang L, Hu LP, Li Q (2024). SSRI antidepressant citalopram reverses the Warburg effect to inhibit hepatocellular carcinoma by directly targeting GLUT1. Cell Rep.

[137] Magagnoli J, Narendran S, Pereira F, Cummings TH, Hardin JW, Sutton SS, Ambati J (2023). Association between fluoxetine use and overall survival among patients with cancer treated with PD-1/L1 immunotherapy. Pharmaceuticals.

[138] Liu W, Stachura P, Xu HC, Umesh Ganesh N, Cox F, Wang R, Lang KS (2020). Repurposing the serotonin agonist tegaserod as an anticancer agent in melanoma: molecular mechanisms and clinical implications. J Exp Clin Cancer Res.

[139] Chan YL, Lai WC, Chen JS, Tseng JTC, Chuang PC, Jou J, Lee CT (2020). TIAM2S mediates serotonin homeostasis and provokes a pro-inflammatory immune microenvironment permissive for colorectal tumorigenesis. Cancers.

[140] Bucsek MJ, Qiao G, MacDonald CR, Giridharan T, Evans L, Niedzwecki B, Liu H (2017). β-adrenergic signaling in mice housed at standard temperatures suppresses an effector phenotype in CD8+ T cells and undermines checkpoint inhibitor therapy. Cancer Res.

[141] Nissen MD, Sloan EK, Mattarollo SR (2018). β-adrenergic signaling impairs antitumor CD8+ T-cell responses to B-cell lymphoma immunotherapy. Cancer Immunol Res.

[142] Qiao G, Bucsek MJ, Winder NM, Chen M, Giridharan T, Olejniczak SH, Hylander BL (2019). β-Adrenergic signaling blocks murine CD8+ T-cell metabolic reprogramming during activation: a mechanism for immunosuppression by adrenergic stress. Cancer Immunol Immunother.

[143] Chen M, Qiao G, Hylander BL, Mohammadpour H, Wang XY, Subjeck JR, Singh AK (2020). Adrenergic stress constrains the development of anti-tumor immunity and abscopal responses following local radiation. Nat Commun.

[144] Qiao G, Chen M, Mohammadpour H, MacDonald CR, Bucsek MJ, Hylander BL, Barbi JJ (2021). Chronic adrenergic stress contributes to metabolic dysfunction and an exhausted phenotype in T cells in the tumor microenvironment. Cancer Immunol Res.

[145] Devi S, Alexandre YO, Loi JK, Gillis R, Ghazanfari N, Creed SJ, Holz LE,
*et al*. Adrenergic regulation of the vasculature impairs leukocyte interstitial migration and suppresses immune responses.
*
Immunity
* 2021, 54: 1219–1230.e1217.. https://doi.org/10.1016/j.immuni.2021.03.025.

[146] Li J, Che M, Zhang B, Zhao K, Wan C, Yang K (2023). The association between the neuroendocrine system and the tumor immune microenvironment: emerging directions for cancer immunotherapy. Biochim Biophys Acta Rev Cancer.

[147] Zhu J, Naulaerts S, Boudhan L, Martin M, Gatto L, Van den Eynde BJ (2023). Tumour immune rejection triggered by activation of α2-adrenergic receptors. Nature.

[148] Thapa S, Cao X (2023). Nervous regulation: beta-2-adrenergic signaling in immune homeostasis, cancer immunotherapy, and autoimmune diseases. Cancer Immunol Immunother.

[149] Fjæstad KY, Rømer AMA, Goitea V, Johansen AZ, Thorseth ML, Carretta M, Engelholm LH (2022). Blockade of beta-adrenergic receptors reduces cancer growth and enhances the response to anti-CTLA4 therapy by modulating the tumor microenvironment. Oncogene.

[150] De Giorgi V, Grazzini M, Benemei S, Marchionni N, Botteri E, Pennacchioli E, Geppetti P (2018). Propranolol for off-label treatment of patients with melanoma. JAMA Oncol.

[151] Kokolus KM, Zhang Y, Sivik JM, Schmeck C, Zhu J, Repasky EA, Drabick JJ (2018). Beta blocker use correlates with better overall survival in metastatic melanoma patients and improves the efficacy of immunotherapies in mice. OncoImmunology.

[152] Mohammadpour H, MacDonald CR, McCarthy PL, Abrams SI, Repasky EA (2021). β2-adrenergic receptor signaling regulates metabolic pathways critical to myeloid-derived suppressor cell function within the TME. Cell Rep.

[153] Zhao X, Li F, Cheng C, Bi M, Li J, Cong J, Wang X (2025). Social isolation promotes tumor immune evasion via β2-adrenergic receptor. Brain Behav Immun.

[154] Singh A, Ranjan A (2023). Adrenergic receptor signaling regulates the CD40-receptor mediated anti-tumor immunity. Front Immunol.

[155] Miao SN, Chai MQ, Liu XY, Wei CY, Zhang CC, Sun NN, Fei QZ (2024). Exercise accelerates recruitment of CD8+ T cell to promotes anti-tumor immunity in lung cancer via epinephrine. BMC Cancer.

[156] Haldar R, Ricon‐Becker I, Radin A, Gutman M, Cole SW, Zmora O, Ben‐Eliyahu S (2020). Perioperative COX2 and β-adrenergic blockade improves biomarkers of tumor metastasis, immunity, and inflammation in colorectal cancer: a randomized controlled trial. Cancer.

[157] Ajmal I, Farooq MA, Duan Y, Yao J, Gao Y, Hui X, Ge Y (2024). Intrinsic ADRB2 inhibition improves CAR-T cell therapy efficacy against prostate cancer. Mol Ther.

[158] Le CP, Nowell CJ, Kim-Fuchs C, Botteri E, Hiller JG, Ismail H, Pimentel MA (2016). Chronic stress in mice remodels lymph vasculature to promote tumour cell dissemination. Nat Commun.

[159] Bruno G, Nastasi N, Subbiani A, Boaretto A, Ciullini Mannurita S, Mattei G, Nardini P (2023). β3-adrenergic receptor on tumor-infiltrating lymphocytes sustains IFN-γ-dependent PD-L1 expression and impairs anti-tumor immunity in neuroblastoma. Cancer Gene Ther.

[160] Geng Q, Li L, Shen Z, Zheng Y, Wang L, Xue R, Xue W (2023). Norepinephrine inhibits CD8+ T-cell infiltration and function, inducing anti-PD-1 mAb resistance in lung adenocarcinoma. Br J Cancer.

[161] Gandhi S, Pandey MR, Attwood K, Ji W, Witkiewicz AK, Knudsen ES, Allen C (2021). Phase I clinical trial of combination propranolol and pembrolizumab in locally advanced and metastatic melanoma: safety, tolerability, and preliminary evidence of antitumor activity. Clin Cancer Res.

[162] Propranolol Hydrochloride and Pembrolizumab in Treating Patients With Stage IIIC–IV Melanoma That Cannot Be Removed by Surgery. ed.: National Library of Medicine (US) 2024.

[163] Naltrexone and Propranolol Combined With Ipilimumab and Nivolumab in Advanced Melanoma. ed.: National Library of Medicine (US) 2024.

[164] SSRI Antidepressant Fluoxetine Improving Immunotherapy Efficacy in Advanced Hepatobiliary Malignancy Patients With Depression and Anxiety: A Randomized Controlled Clinical Trial. ed.: National Library of Medicine (US) 2025.

[165] Cox MA (2024). Adrenergic signaling dampens T cell activity during chronic infection and cancer. Trends Neuroscis.

[166] Dimitrov S, Lange T, Born J (2010). Selective mobilization of cytotoxic leukocytes by epinephrine. J Immunol.

[167] Farooq MA, Ajmal I, Hui X, Chen Y, Ren Y, Jiang W (2023). β2-adrenergic receptor mediated inhibition of T cell function and Its implications for CAR-T cell therapy. Int J Mol Sci.

[168] Asp M, Bergenstråhle J, Lundeberg J (2020). Spatially resolved transcriptomes-next generation tools for tissue exploration. BioEssays.

[169] Du Y, Ding X, Ye Y (2024). The spatial multi-omics revolution in cancer therapy: precision redefined. Cell Rep Med.

[170] Chen MM, Gao Q, Ning H, Chen K, Gao Y, Yu M, Liu CQ (2025). Integrated single-cell and spatial transcriptomics uncover distinct cellular subtypes involved in neural invasion in pancreatic cancer. Cancer Cell.

[171] Moffitt JR, Bambah-Mukku D, Eichhorn SW, Vaughn E, Shekhar K, Perez JD, Rubinstein ND (2018). Molecular, spatial, and functional single-cell profiling of the hypothalamic preoptic region. Science.

[172] Zhang Y, Chen K, Sloan SA, Bennett ML, Scholze AR, O′Keeffe S, Phatnani HP (2014). An RNA-sequencing transcriptome and splicing database of glia, neurons, and vascular cells of the cerebral cortex. J Neurosci.

[173] Liu S, Iorgulescu JB, Li S, Borji M, Barrera-Lopez IA, Shanmugam V, Lyu H (2022). Spatial maps of T cell receptors and transcriptomes reveal distinct immune niches and interactions in the adaptive immune response. Immunity.

[174] Hickey JW, Agmon E, Horowitz N, Tan TK, Lamore M, Sunwoo JB, Covert MW (2024). Integrating multiplexed imaging and multiscale modeling identifies tumor phenotype conversion as a critical component of therapeutic T cell efficacy. Cell Syst.

[175] Bernstein JG, Boyden ES (2011). Optogenetic tools for analyzing the neural circuits of behavior. Trends Cogn Sci.

[176] Schiller M, Azulay-Debby H, Boshnak N, Elyahu Y, Korin B, Ben-Shaanan TL, Koren T (2021). Optogenetic activation of local colonic sympathetic innervations attenuates colitis by limiting immune cell extravasation. Immunity.

[177] Ben-Shaanan TL, Schiller M, Azulay-Debby H, Korin B, Boshnak N, Koren T, Krot M (2018). Modulation of anti-tumor immunity by the brain’s reward system. Nat Commun.

[178] Park HJ, Lee SC, Park SH (2024). Norepinephrine stimulates M2 macrophage polarization via β2-adrenergic receptor-mediated IL-6 production in breast cancer cells. Biochem Biophys Res Commun.

[179] Szabo PA, Levitin HM, Miron M, Snyder ME, Senda T, Yuan J, Cheng YL (2019). Single-cell transcriptomics of human T cells reveals tissue and activation signatures in health and disease. Nat Commun.

[180] Le J, Dian Y, Zhao D, Guo Z, Luo Z, Chen X, Zeng F (2025). Single-cell multi-omics in cancer immunotherapy: from tumor heterogeneity to personalized precision treatment. Mol Cancer.

[181] Gao Y, Dong K, Gao Y, Jin X, Yang J, Yan G, Liu Q (2024). Unified cross-modality integration and analysis of T cell receptors and T cell transcriptomes by low-resource-aware representation learning. Cell Genomics.

[182] Engblom C, Thrane K, Lin Q, Andersson A, Toosi H, Chen X, Steiner E (2023). Spatial transcriptomics of B cell and T cell receptors reveals lymphocyte clonal dynamics. Science.

[183] Benotmane JK, Kueckelhaus J, Will P, Zhang J, Ravi VM, Joseph K, Sankowski R (2023). High-sensitive spatially resolved T cell receptor sequencing with SPTCR-seq. Nat Commun.

[184] Rodriques SG, Stickels RR, Goeva A, Martin CA, Murray E, Vanderburg CR, Welch J (2019). Slide-seq: a scalable technology for measuring genome-wide expression at high spatial resolution. Science.

[185] Wang Z, Zhao Y, Zhang L (2024). Emerging trends and hot topics in the application of multi-omics in drug discovery: a bibliometric and visualized study. Curr Pharm Anal.

[186] Liu Y, Zhang S, Liu K, Hu X, Gu X (2024). Advances in drug discovery based on network pharmacology and omics technology. Curr Pharm Anal.

[187] Singh J, Luqman S, Meena A (2023). Carvacrol as a prospective regulator of cancer targets/signalling pathways. Curr Mol Pharmacol.

[188] Lin A, Xiong M, Jiang A, Huang L, Wong HZH, Feng S, Zhang C (2025). The microbiome in cancer. iMeta.

[189] Gao Z, Jiang A, Li Z, Zhu L, Mou W, Shen W, Luo P (2025). Heterogeneity of intratumoral microbiota within the tumor microenvironment and relationship to tumor development. Med Res.

[190] Lin A, Ye P, Li Z, Jiang A, Liu Z, Cheng Q, Zhang J (2025). Natural killer cell immune checkpoints and their therapeutic targeting in cancer treatment. Research.

[191] Zhou CB, Zhou YL, Fang JY (2021). Gut microbiota in cancer immune response and immunotherapy. Trends Cancer.

[192] Mafe AN, Büsselberg D (2025). Modulation of the neuro–cancer connection by metabolites of gut microbiota. Biomolecules.

[193] Nobels A, van Marcke C, Jordan BF, Van Hul M, Cani PD (2025). The gut microbiome and cancer: from tumorigenesis to therapy. Nat Metab.

[194] Yousefi Y, Baines KJ, Maleki Vareki S (2024). Microbiome bacterial influencers of host immunity and response to immunotherapy. Cell Rep Med.

[195] Luo Y, Sun L, Peng Y (2025). The structural basis of the G protein–coupled receptor and ion channel axis. Curr Res Struct Biol.

[196] Gonzalez-Hernandez AJ, Munguba H, Levitz J (2024). Emerging modes of regulation of neuromodulatory G protein-coupled receptors. Trends Neuroscis.

[197] Pin JP, Bettler B (2016). Organization and functions of mGlu and GABAB receptor complexes. Nature.

[198] Bodzęta A, Scheefhals N, MacGillavry HD (2021). Membrane trafficking and positioning of mGluRs at presynaptic and postsynaptic sites of excitatory synapses. Neuropharmacology.

[199] Park JC, Luebbers A, Dao M, Semeano A, Nguyen AM, Papakonstantinou MP, Broselid S (2023). Fine-tuning GPCR-mediated neuromodulation by biasing signaling through different G protein subunits. Mol Cell.

